# Isocyanide Reactions Toward the Synthesis of 3-(Oxazol-5-yl)Quinoline-2-Carboxamides and 5-(2-Tosylquinolin-3-yl)Oxazole

**DOI:** 10.3389/fchem.2019.00433

**Published:** 2019-06-14

**Authors:** Zahra Yasaei, Zeinab Mohammadpour, Morteza Shiri, Zahra Tanbakouchian, Shima Fazelzadeh

**Affiliations:** Department of Chemistry, Faculty of Physics and Chemistry, Alzahra University, Tehran, Iran

**Keywords:** palladium acetate, carboxamidation, isocynides, sulfonylation, TosMIC

## Abstract

A palladium-catalyzed three-component reaction between 5-(2-chloroquinolin-3-yl) oxazoles, isocyanides, and water to yield 3-(oxazol-5-yl)quinoline-2-carboxamides is described. Interestingly, sulfonylation occurred when the same reaction was performed with toluenesulfonylmethyl isocyanide (TosMIC) as an isocyanide source. The reaction with 5-(2-chloroquinolin-3-yl)oxazoles and TosMIC in the presence of Cs_2_CO_3_ in DMSO afforded 5-(2-Tosylquinolin-3-yl)oxazoles. In basic media, TosMIC probably decomposed to generate Ts^−^ species, which were replaced with Cl^−^. Tandem oxazole formation with subsequent sulfonylation of 2-chloroquinoline-3-carbaldehydes to form directly 5-(2-tosylquinolin-3-yl)oxazoles was also investigated.

## Introduction

Quinolines are heterocyclic compounds exhibiting diverse and well-documented bioactivity and physical properties as well as existing as scaffolds in complex structures of natural products (Michael, 2002; Hranjec et al., 2017). Accordingly, quinoline synthesis, and functionalization has attracted much attention from synthetic organic chemists (Marco-Contelles et al., 2009; Shiri et al., 2011; Prajapati et al., 2014; Sharma et al., 2018; Nainwal et al., 2019).

However, isocyanides play an important role in synthesizing *N*-containing heterocycles and are particularly widely applied in Ugi and Passirini reactions (Domling and Ugi, 2000; Domling, 2006). Amides are especially valuable as precursors in synthesizing of bioactive and natural structures, in medicinal chemistry as well as protein synthesis (Bode, 2006; Rönn et al., 2008).

**Figure 1:**
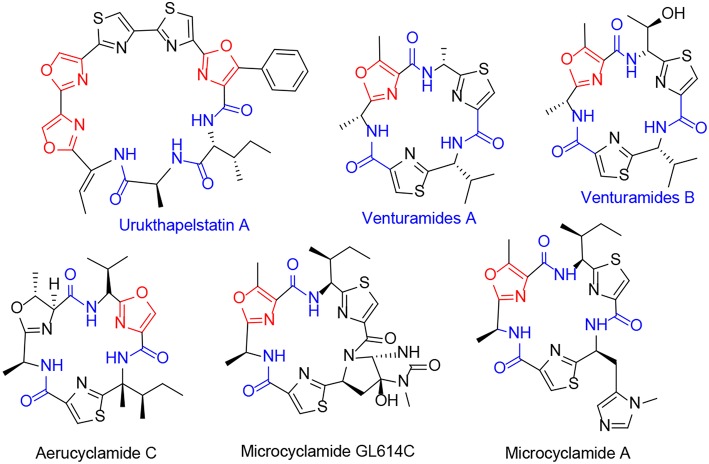
Several natural products with amide and oxazole moieties.

Many natural products such as urukthapelstatin A have been isolated from marine sources, these contains, several aminocarbonyl and oxazole functional groups with cytotoxic activity against human lung cancer (Yu et al., 2009). Similarly, venturamides A and B showed *in vitro* antimalarial activity (Linington et al., 2007; Davyt and Serra, 2010). Additionally, aerucyclamid C, a hexameric cyclopeptide, extremely active against *T. brucei rhodesiense*, which causes sleeping sickness, and exhibits lower activity against *P. falciparum*, the deadliest species of *Plasmodium*, which causes malaria (Davyt and Serra, 2010). Microcyclamide A is a cyclic hexapeptide with three five-membered heterocycles. It is isolated from cyanobacterium *M. aeruginosa*, and it has exhibited cytotoxic effects against P388 murine leukemia (Ishida et al., 2000; Davyt and Serra, 2010; Raveh et al., 2010) (Figure 1).

Classical procedures for amide bond formation include reactions between carboxylic acids and amines (Linington et al., 2007; Davyt and Serra, 2010). Another route involves reactions of acyl halides, acyl azides, acyl imidazoles, anhydrides, or esters (activated carboxylic acid species) with amines (Ulijn et al., 2002; Montalbetti and Falque, 2005). As well as classical routes for amides synthesis which have own merits and demerits, direct aminocarbonylation from aryl halides in metal-catalyzed reactions has attracted attention from chemists due to its significant advantages benzamide preparation (Åkerbladh et al., 2017). In this regard, many synthetic methods have been introduced, including copper-catalyzed reactions of aryl halides and isocyanides in DMSO (Yavari et al., 2014). A palladium-catalyzed reaction was developed for amidation of aryl halides (Jiang et al., 2011), as well as the synthesis of 4-aminophthalazin-1(*2H*)-ones in a palladium-catalyzed reaction with isocyanide insertion in a multi-component reaction, which is difficult to achieve via a classical route (Vlaar et al., 2011). Palladium-catalyzed isocyanide insertion was applied to a carboxamidation/hydroamidation reaction to synthesize isoindolin-1-one derivatives (Pathare et al., 2016). Another example is the synthesis of isoquinolin-1(*2H*)-one derivatives via a palladium-catalyzed cascade reaction from isocyanide and amides (Wang et al., 2002; Tyagi et al., 2012; Chaudhary et al., 2013). Very recently, Guan et al reported an efficient method for the synthesis of multisubstituted 1*H*-imidazo-[4,5-*c*]quinoline derivatives via sequential van Leusen/Staudinger/aza-Wittig/carbodiimide-mediated cyclization (Guan et al., 2018).

## Materials and Methods

### General

The solvents and chemicals purchased from Merck and Aldrich chemical companies. Unless otherwise mentioned they used without further purification. Melting points are taken on an Electrothermal 9100 apparatus and are uncorrected. IR spectras recorded on a Shimadzu Infra-Red Spectroscopy IR-435. Nuclear magnetic resonance (NMR) spectra recorded on a Bruker AVANCE Spectrometer (400 MHz for ^1^H, 100 MHz for ^13^C) in DMSO-d_6_ and CDCl_3_ as solvent, TMS used as internal standard. The elemental analysis carried out with a Leco CHNS model 932. Mass spectra recorded on Agilent Technology (HP) 5973 Network Mass Selective Detector operating at an ionization potential of 70 eV.

#### The Typical Procedure for the Synthesis of 5-(2-chloroquinolin-3-yl)oxazole 3a

A mixture of 2-chloro-quinoline-3-carbaldehyde **2a** (191 mg, 1.0 mmol), *p*-toluenesulfonylmethyl isocyanide **1** (234 mg, 1.2 mmol) and K_2_CO_3_ (341 mg, 2.5 equiv.) was added to EtOH (5.0 ml) and stirred for 3.5 h, at room temperature. After completion of the reaction, monitored by TLC, the mixture poured into cool water and stirred for 30 min. The product **3a** filtered, washed with water two times and dried on the air.

#### The Typical Procedure for the Synthesis of N-cyclohexyl-3-(oxazol-5-yl)quinoline-2-carboxamide 5a

A mixture of 5-(2-chloroquinolin-3-yl)oxazole **3a** (230 mg, 1.0 mmol), of Pd(OAc)_2_ (11 mg, 5 mol%) and Cs_2_CO_3_ (325 mg, 1.0 mmol) stirred in DMSO:H_2_O, 9:1 (5 mL) at 80°C for 15 min. Cyclohexyl isocyanide **4a** (120 mg, 1.1 mmol) was added and the reaction stirred for 4 h. After completion of the reaction (the progress of the reaction was monitored by TLC) organic layer was extracted by DCM, washed with brine, dried over Na_2_SO_4_ and its solvent evaporated on a rotary evaporator. The residue was washed with 2-propanol and recrystallized in methanol to give **5a**. It is noteworthy that **5a** and **5h** purified by washing with 2-propanol but other derivatives were purified by a column chromatography (*n*-hexan: ethyl acetate 3:1) and recrystallized in EtOH.

#### The Typical Procedure for the Synthesis of 5-(2-tosylquinolin-3-yl)oxazole 6a-e

A mixture of 2-chloro-quinoline-3-carbaldehyde **2a** (191 mg, 1.0 mmol), *p*-toluenesulfonylmethyl isocyanide **1** (468 mg, 2.4 mmol) and Cs_2_CO_3_ (810 mg, equiv.) was added to DMSO (5.0 ml) and stirred for 5 h at 80°C. After completion of the reaction and monitored by TLC, the mixture poured into cool water and stirred for 30 min, then extracted with DCM. The product **6a** purified by a column chromatography (*n*-hexan: ethyl acetate 4:1).

### Supplementary Material

#### N-Cyclohexyl-3-(oxazol-5-yl) quinolone-2-carboxamide (5a)

Copies of NMR spectra are provided as Supplementary Materials. White powder, mp.: 123–128°C. ^**1**^**H-NMR** (400 MHz, CDCl_3_): δ = 1.25–1.52 (m, 5H), 1.68–171 (m, 1H), 1.80–1.85 (m, 2H), 2.10–213 (m, 2H), 3.99–4.02 (m, 1H), 7.60 (s, 1H), 7.67 (t, *J* = 8.0 Hz, 2H), 7.82 (t, *J* = 7.8 Hz, 1H), 7.90 (d, *J* = 8.4 Hz, 1H), 8.03 (s, 1H), 8.14 (d, *J* = 8.4 Hz, 1H), 8.44 (s, 1H) ppm. ^**13**^**C-NMR** (100 MHz, CDCl_3_): δ = 25.0, 25.6, 33.0, 48.6, 120.4, 125.9, 127.9, 128.0, 128.6, 129.5 131.0, 137.9, 145.9, 148.5, 148.8, 151.0, 164.3 ppm. **Mass**: m/z 321 (M+) (calcd. For C_19_H_19_N_3_O_2_: 321.37). FT-IR (KBr): ν_max_: 1604, 3444 cm^−1^. **Anal. calcd. for** C_19_H_19_N_3_O_2_: C, 71.01; H, 5.96; N, 13.08. Found: C,71.10; H,5.81; N,13.17.

#### N-Cyclohexyl-8-methyl-3-(oxazol-5-yl)quinolone-2-carboxamide (5b)

White powder, mp: 181–185°C. ^**1**^**H-NMR** (400 MHz, DMSO-d_6_): δ = 1.15–1.74 (m, 10H), 2.75 (s, 3H), 3.83 (m, 1H), 7.52 (s, 1H), 7.63 (d, *J* = 8.0 Hz, 1H), 7.73 (d, *J* = 6.8 Hz, 1H), 7.97 (d, *J* = 8.0 Hz, 1H), 8.60 (s, 1H), 8.74 (s, 1H) ppm. ^**13**^**C-NMR** (100 MHz, DMSO-d_6_): δ = 17.8, 25.0, 25.6, 25.8, 32.5, 33.8, 48.5, 118.6, 121.8, 125.0, 126.7, 127.6, 128.3, 131.4, 135.2, 136.9, 145.0, 147.8, 153.0, 167.0 ppm. **Mass**: m/z 335 (M^+^) (calcd. for C_20_H_21_N_3_O_2_: 335.40). FT-IR (KBr): ν_max_: 1646, 2853, 2922, 3310, 3444 cm^−1^. Anal. calcd. for C_20_H_21_N_3_O_2_: C, 71.62; H, 6.31; N, 12.53%. Found: C, 71.55; H, 6.47; N, 12.42%.

#### N-Butyl-6-methyl-3-(oxazol-5-yl)quinoline-2-carboxamide (5c)

White powder, mp: 124–127°C. ^**1**^**H-NMR** (400 MHz, CDCl_3_): δ = 1.01 (t, *J* = 7.2 Hz, 3H), 1.45–1.54 (m, 2H), 1.67–1.74 (m, 2H), 2.61 (s, 3H), 3.53 (dd, *J* = 13.4 Hz, *J* = 6.8 Hz, 2H), 7.60 (s, 1H), 7.65 (s, 1H), 7.68 (s, 1H), 7.77 (s, 1H), 8.03 (t, *J* = 5.2 Hz, 2H), 8.37 (s, 1H) ppm. ^**13**^**C-NMR** (100 MHz, CDCl_3_): δ = 13.8, 20.2, 21.8, 31.7, 39.5, 120.4, 125.7, 125.8, 126.5, 126.6, 128.1, 129.0, 133.4, 137.2, 138.9, 142.9, 150.9, 165.2 ppm. **Mass**: m/z 309 (M^+^) (calcd for C_18_H_19_N_3_O_2_: 309.36). FT-IR (KBr): ν_max_: 1542, 1652, 2858, 2928, 2956, 2922, 3114, 3299 cm^−1^. **Anal. calcd. for**: C_18_H_19_N_3_O_2_, C, 69.88; H, 6.19; N, 13.58%. Found C, 67.03; H, 6.24; N, 13.65%.

#### N-Cyclohexyl-6-methoxy-3-(oxazol-5-yl)quinoline-2-carboxamide (5d)

White powder, mp: 157–162°C. ^**1**^**H-NMR** (400 MHz, DMSO-d_6_): δ = 1.04–1.27 (m, 5H), 1.57–1.74 (m, 5H), 3.93(s, 3H), 3.93 (m, 1H), 7.52 (dd, *J* = 9.2, *J* = 2.8 Hz, 1H), 7.61 (d, *J* = 2.8 Hz, 1H), 7.92 (d, *J* = 9.2 Hz, 1H), 7.96 (s, 1H), 8.71 (s, 1H), 8.77 (s,1H) ppm. ^**13**^**C-NMR** (100 MHz, DMSO-d_6_): δ = 24.9, 25.8, 30.8, 31.2, 33.8, 47.9, 56.2, 106.7, 115.8, 121.3, 124.6, 127.5, 128.3, 129.6, 135.9, 142.4, 143.1, 146.5, 153.4, 158.7 ppm. **Mass**: m/z 351 (M+) (calcd. for C_20_H_21_N_3_O_3_:351.40). **FT-IR** (KBr): ν_max_: 1666, 2927, 2966, 3286, 3423 cm^−1^. **Anal. calcd. for** C_20_H_21_N_3_O_3_: C, 68.36; H, 6.02; N, 11.96%. Found: C, 68.47; H, 6.14; N, 12.11%.

#### N-Cyclohexyl-3-(oxazol-5-yl)benzo[h]quinoline-2-carboxamide (5e)

White powder, mp: 224–267°C. ^**1**^**H-NMR** (400 MHz, CDCl_3_): δ = 1.28–1.58 (m, 6H), 1.85–1.88 (m, 2H), 2.16–2.19 (m, 2H), 4.07- 4.10 (m, 1H), 7.30 (s, 1H), 7.74 (d, *J* = 2.4 Hz, 1H), 7.79-7.85 (m, 3H), 7.93 (d, *J* = 8.8 Hz, 1H), 7.97 (d, *J* = 7.6 Hz, 1H), 8.07 (s, 1H), 8.49 (s, 1H), 9.19 (d, *J* = 7.6 Hz, 1H) ppm. ^**13**^**C-NMR** (100 MHz, CDCl_3_): δ = 24.9, 25.7, 29.7, 33.2, 48.5, 77.2, 121.3, 124.3, 124.7, 126.6, 126.9, 127.7, 128.2, 129.1, 130.1, 130.6, 134.1, 137.5, 144.2, 146.7, 148.5, 151.1, 164.5 ppm. **Mass**: m/z 371 (M^+^) (calcd for C_23_H_21_N_3_O_2_:371.43). **FT-IR** (KBr): ν_max_: 1646, 2852, 2936, 3119, 3294, 3448 cm^−1^. **Anal. calcd. for**: C_23_H_21_N_3_O_2_, C, 74.37; H, 5.70; N, 11.31%. Found: C, 74.29; H, 5.84; N, 11.52%.

#### 6-Chloro-N-cyclohexyl-3-(oxazol-5-yl)quinoline-2-carboxamide (5f)

White powder, mp: 226–232°C. ^**1**^**H-NMR** (400 MHz, CDCl_3_): δ = 1.25–1.52 (m, 5H), 1.68–1.83 (m, 3H), 2.09–2.12 (m, 2H), 3.94–4.04 (m, 1H), 7.54 (d, *J* = 8.0 Hz, 1H), 7.63 (s, 1H), 7.71 (dd, *J* = 9.0 Hz, *J* = 2.4 Hz, 1H), 7.86 (d, *J* = 2.4 Hz, 1H), 8.02 (s, 1H), 8.05 (d, *J* = 9.2 Hz, 1H), 8.34 (s, 1H) ppm. ^**13**^**C-NMR** (100 MHz, CDCl_3_): δ = 24.9, 25.6, 33.0, 48.7, 77.3, 121.2, 126.4, 126.6, 128.5, 131.0, 131.9, 134.5, 136.3, 144.1, 147.9, 149.0, 151.2, 164.2 ppm. **Mass**: m/z 355 (M+) (calcd. for C_19_H_18_ ClN_3_O_2_: 355.82). **FT-IR** (KBr): ν_max_: 663, 1649, 2852, 2923, 3279, 3443 cm^−1^. Anal. calcd. for: C_19_H_18_ClN_3_O_2_, C, 64.13; H, 5.10; N, 11.81%. Found: C, 64.20; H, 5.16; N, 11.70%.

#### N-(tert-Butyl)-6-chloro-3-(oxazol-5-yl)quinoline-2-carboxamide (5g)

White powder, mp: 190–197°C. ^**1**^**H-NMR** (400 MHz, DMSO-d_6_): δ = 1.43 (s, 9H), 7.75 (s, 1H), 7.86 (dd, *J* = 9.2 Hz, *J* = 2.4 Hz, 1H), 8.14 (d, *J* = 8.8 Hz, 1H), 8.28 (d, *J* = 2.4 Hz, 1H), 8.46 (s, 1H), 8.64 (s, 1H), 8.76 (s, 1H) ppm. ^**13**^**C-NMR** (100 MHz, DMSO-d_6_): δ = 28.8, 51.5, 119.5, 125.5, 127.4, 128.3, 131.3, 131.8, 132.7, 133.8, 144.5, 147.4, 153.4, 167.1 ppm. **Mass**: m/z 329.78 (M+) (calcd. for C_17_H_16_N_3_O_2_: 329.09). FT-IR (KBr): ν_max_: 1626, 2852, 2928, 3334, 3423 cm^−1^. Anal. calcd. for C_17_H_16_ ClN_3_O_2_: C, 61.91; H, 4.89; N, 12.74. Found: C, 62.04; H, 4.95; N, 12.89.

#### N-(tert-Butyl)-3-(oxazol-5-yl)quinolone-2-carboxamide (5h)

White powder, mp: 102–106°C. ^**1**^**H-NMR** (400 MHz, DMSO-d_6_): δ = 1.43 (s, 9H), 7.56 (s, 1H), 7.72 (t, *J* = 7.4 Hz, 1H), 7.87 (t, *J* = 7.6 Hz, 1H), 8.13 (t, *J* = 8.8 Hz, 2H), 8.41 (s, 1H), 8.61(s, 1H), 8.77 (s, 1H) ppm. ^**13**^**C-NMR** (100 MHz, DMSO-d_6_): δ = 28.8, 51.4, 118.7, 125.1, 127.5, 128.4, 128.8, 129.1, 131.4, 134.7, 146.0, 147.8, 153.0, 153.1, 167.4 ppm. **Mass**: m/z 295 (M+) (calcd. for C_17_H_17_N_3_O_2_: 295.34). **FT-IR** (KBr): ν_max_: 1658, 2904, 2966, 3422 cm^−1^. **Anal. calcd. for** C_17_H_17_N_3_O_2_: C, 69.14; H, 5.80; N, 14.23%. Found: C, 69.20; H, 5.87; N, 14.35%.

#### 5-(2-Tosylquinolin-3-yl)oxazole (6a)

White powder, mp: 149–152°C. ^1^H-NMR (400 MHz, DMSO-d_6_): δ = 2.46 (s, 3H), 7.50 (d, *J* = 7.2 Hz, 2H), 7.77–7.90 (m, 6H), 8.18 (d, *J* = 7.6 Hz, 1H), 8.69 (s, 1H), 8.92 (s, 1H) ppm. ^13^C-NMR (100 MHz, DMSO-d_6_): δ = 21.6, 118.2, 127.6, 128.2, 128.9, 129.5, 129.6, 130.1, 130.6, 132.9, 135.5, 141.1, 144.9, 145.3, 145.6, 153.4, 155.1 ppm. Mass: m/z 350 (M^+^) (calcd for C_19_H_14_N_2_O_3_S:350.39). FT-IR (KBr): ν_max_: 683, 1073, 1103, 2851, 2920 cm^−1^. Anal. calcd. for: C_19_H_14_N_2_O_3_S, C, 65.13; H, 4.03; N, 7.99; S, 9.15%. Found: C, 65.23; H, 4.14; N, 8.06; S, 9.21%.

#### 5-(8-Methyl-2-tosylquinolin-3-yl)oxazole (6b)

White powder, mp: 162–167°C. ^1^H-NMR (400 MHz, DMSO-d_6_): δ = 2.18 (s, 3H), 2.50 (s, 3H), 7.55 (d, *J* = 8.0 Hz, 1H), 7.71 (d, *J* = 6.8 Hz, 2H), 7.88 (s, 1H), 7.90 (d, *J* = 7.6 Hz, 2H), 8.03 (dd, *J* = 6.8 Hz, *J* = 2.8 Hz, 1H), 8.73 (s, 1H), 8.95 (s, 1H) ppm. ^13^C-NMR (100 MHz, DMSO-d_6_): δ = 16.5, 21.7, 117.5, 126.6, 127.7, 128.3, 129.9, 130.1, 130.4, 132.5, 135.3, 137.4, 140.3, 143.5, 145.3, 145.5, 153.6, 153.8 ppm. Mass: m/z 364 (M^+^) (calcd for C_20_H_16_N_2_O_3_S:364.42). FT-IR (KBr): ν_max_: 1294, 1379, 2852, 2922 cm^−1^. Anal. calcd. for: C_20_H_16_N_2_O_3_S, C, 65.92; H, 4.43; N, 7.69; S, 8.80%. Found: C, 66.03; H, 4.49; N, 7.78; S, 8.93%.

#### 5-(6-Methyl-2-tosylquinolin-3-yl)oxazole (6c)

White powder, mp: 124–127°C. ^1^H-NMR (400 MHz, CDCl_3_): δ = 2.51 (s, 3H), 2.59 (s, 3H), 7.30 (s, 1H), 7.38 (d, *J* = 6.4 Hz, 2H), 7.62 (d, *J* = 8.4 Hz, 1H), 7.68 (s, 1H), 7.84 (d, *J* = 8.0 Hz, 1H), 7.89 (d, *J* = 7.6 Hz, 2H), 8.10 (s, 1H), 8.44 (s, 1H) ppm. ^13^C-NMR (100 MHz, DMSO-d_6_): δ = 21.6, 21.7, 118.3, 127.3, 127.5, 128.2, 129.2, 129.5, 130.0, 135.1, 135.7, 140.2, 140.8, 142.9, 143.6, 145.2, 145.7, 153.3 ppm. Mass: m/z 364 (M^+^) (calcd for C_20_H_16_N_2_O_3_S:364.42). FT-IR (KBr): ν_max_: 823, 1375, 2853, 2923 cm^−1^. Anal. calcd. for: C_20_H_16_N_2_O_3_S, C, 65.92; H, 4.43; N, 7.69; S, 8.80%. Found: C, 65.98; H, 4.49; N, 7.77; S, 8.86%.

#### 5-(6-Methoxy-2-tosylquinolin-3-yl)oxazole (6d)

White powder, mp: 187–190°C. ^1^H-NMR (400 MHz, CDCl_3_): δ = 2.51 (s, 3H), 3.99 (s, 3H), 7.16 (d, *J* = 8.8 Hz, 2H), 7.30 (s, 1H), 7.39 (s, 1H), 7.58 (d, *J* = 8.8 Hz, 2H), 7.84 (d, *J* = 9.2 Hz, 2H), 7.90 (d, *J* = 8.4 Hz, 1H), 8.10 (s, 1H), 8.42 (s, 1H) ppm. ^13^C-NMR (100 MHz, CDCl_3_): δ = 37.1, 55.8, 104.6, 119.1, 124.0, 124.4, 124.8, 129.2, 129.6, 131.6, 135.6, 137.5, 138.4, 138.5, 144.5, 147.1, 147.6, 147.7 ppm. Mass: m/z 380 (M^+^) (calcd for C_20_H_16_N_2_O_4_S: 380.42). FT-IR (KBr): ν_max_: 1037, 117, 2850, 2920 cm^−1^. Anal. calcd. for: C_20_H_16_N_2_O_4_S, C, 63.14; H, 4.24; N, 7.36; S, 8.43%. Found: C, 63.21; H, 4.29; N, 7.43; S, 8.55%.

#### 5-(2-Tosylbenzo[h]quinolin-3-yl)oxazole (6e)

White powder, mp: 202–207°C. ^1^H-NMR (400 MHz, CDCl_3_): δ = 2.60 (s, 3H), 7.30 (s, 1H), 7.52 (d, *J* = 8.0 Hz, 2H), 7.57 (t, *J* = 7.2 Hz, 1H), 7.70 (t, *J* = 6.8 Hz, 1H), 7.73 (d, *J* = 8.8 Hz, 1H), 7.89 (d, *J* = 8.4 Hz, 1H), 7.92 (d, *J* = 9.2 Hz, 1H), 8.04 (d, *J* = 8.4 Hz, 2H), 8.16 (d, *J* = 5.6 Hz, 1H), 8.20 (d, *J* = 8.4 Hz, 1H), 8.60 (s, 1H) ppm. ^13^C-NMR (100 MHz, CDCl_3_): δ = 21.8, 119.0, 124.1, 124.4, 127.0, 127.8, 128.0, 128.7, 129.3, 129.3, 130.2, 130.5, 131.1, 134.0, 135.3, 137.5, 134.3, 144.8, 145.5, 151.6, 153.3 ppm. Mass: m/z 400 (M^+^) (calcd for C_23_H_16_N_2_O_3_S: 400.45). FT-IR (KBr): ν_max_: 1313, 1400, 2853, 2920 cm^−1^. Anal. calcd. for: C_23_H_16_N_2_O_3_S, C, 68.98; H, 4.03; N, 7.00; S, 8.01%. Found: 69.06; H, 4.11; N, 7.12; S, 8.14%.

## Results and Discussion

In continuation of our interest in quinoline chemistry (Shiri et al., 2012, 2016a, 2017a) and isocyanide reactions (Shiri et al., 2016b, 2017b; Salehi and Shiri, 2019), we began our investigation with 2-chloroquinoline-3-carbaldehyde (**1**) and its two step reaction with two different isocyanides. In the presence of K_2_CO_3_, 2-chloroquinoline-3-carbaldehyde (**1**) and 4-toluenesulfonylmethyl isocyanide (TosMIC) (**2**) furnished 5-(2-chloroquinolin-3-yl)oxazole (**3a**). Several 5-(2-chloroquinolin-3-yl)oxazoles (**3**) were prepared under the same conditions. The reaction of quinoline **3a** with cyclohexyl isocyanide **4a** was selected as a model reaction in the presence of Pd(OAc)_2_, Ph_3_P, and Cs_2_CO_3_, in 1,4-dioxane with a few drops of H_2_O as solvent at 80 °C. Desired product **5a** was obtained in 86% yield (Table 1). Solvent screening showed that DMSO is the best solvent (Table 1, entry 6). Other Pd sources such as Pd(PPh_3_)_4_ and PdCl_2_ did not improve the product yield, however, the best yield was obtained with 5 mol% of Pd(OAc)_2_ even without PPh_3_ (Table 1, entries 7–9). Without palladium, the reaction did not occur (Table 1, entry 10). Moreover, the effect of base is crucial for reaction completion. Hence, different bases were investigated, including K_2_CO_3_, NaOAc, (CH_3_)_3_OK, DABCO, and Et_3_N (Table 1, entries 14–18). In this survey, it was found that increasing the temperatures or the reaction time decreased the product yield.

**Table 1:** Optimization of the reaction condition for the synthesis of *N*-cyclohexyl-3-(oxazol-5-yl)quinoline-2-carboxamide **5a** from **3a**.

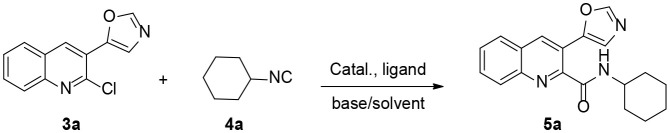					
**Entry**	**Solvent**	**Base**	**Catalyst/Ligand**	**Time(h)**	**Yield 5a**^**a**^ **(%)**
**1**	Dioxane	Cs_2_CO_3_	Pd(OAc)_2_/PPh_3_	8	86
**2**^**c**^	CH_3_CN	Cs_2_CO_3_	Pd(OAc)_2_/PPh_3_	8	21
**3**^**c**^	EtOH	Cs_2_CO_3_	Pd(OAc)_2_/PPh_3_	8	0
**4**	Toluene	Cs_2_CO_3_	Pd(OAc)_2_/PPh_3_	8	5
**5**	DMF	Cs_2_CO_3_	Pd(OAc)_2_/PPh_3_	8	82
**6**	DMSO	Cs_2_CO_3_	Pd(OAc)_2_/PPh_3_	6	92
**7**	DMSO	Cs_2_CO_3_	Pd(OAc)_2_/-	1.5	92
**8**	DMSO	Cs_2_CO_3_	PdCl_2_	12	74
**9**	DMSO	Cs_2_CO_3_	Pd(PPh_3_)_3_	12	61
**10**	DMSO	Cs_2_CO_3_	−	12	0
**11**	DMSO	K_2_CO_3_	Pd(OAc)_2_/−	12	90
**12**	DMSO	NaOAc	Pd(OAc)_2_/−	12	20
**13**	DMSO	KO^t^Bu	Pd(OAc)_2_/−	12	48
**14**	DMSO	DABCO	Pd(OAc)_2_/−	12	5
**15**	DMSO	Et_3_N	Pd(OAc)_2_/−	12	0

a *Isolated yields*.

^b^All reactions were carried out using **3a** (1 mmol), **4a** (1.1 mmol), catalyst (5 mol %), base (1 mmol), and solvent (2.0 mL) and 80°C unless otherwise noted.

c *At reflux*.

With the optimized reaction conditions in hand (Pd(OAc)_2_ (5 mol%), Cs_2_CO_3_ (1 equiv.), DMSO + H_2_O (9:1), 80°C), the generality of the reaction was explored (Table 2). A range of quinolines **3** bearing electron-donating groups, such as Me, and OMe and electron-withdrawing groups, such as Cl and benzo, reacted with cyclohexyl isocyanide and *n*-butylisocyanide to afford the corresponding 3-(oxazol-5-yl)quinoline-2-carboxamides **5a-f** in 70%-94% yields (Table 2). Moreover, the bulky *tert*-butyl isocyanide smoothly participated in this reaction to furnish **5g** and **5h** in 67 and 76% yield, respectively. The yield with 1,1,3,3-tetramethylbutyl isocyanide was too low to allow its isolation and characterization.

**Table 2:** Synthesis of various derivatives of **5a**-**5h**^a^.

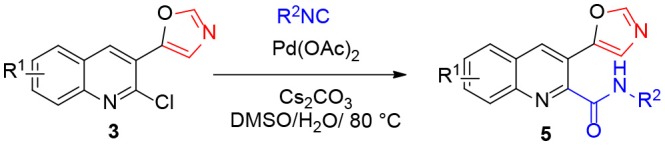
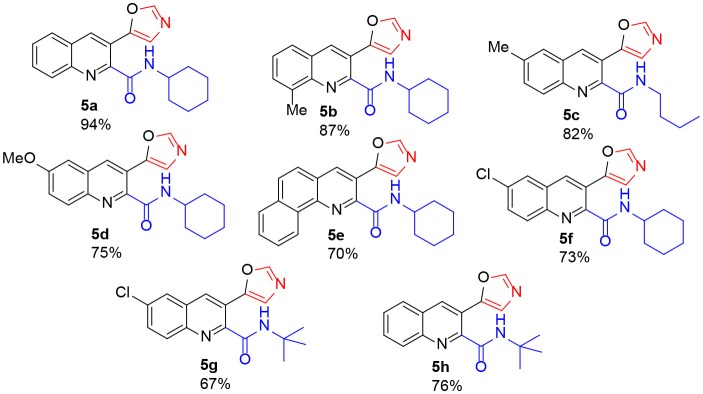

a *All reactions were performed using **3** (1 mmol), **4** (1.1 mmol), Pd(OAc)_2_ (5 mol%), Cs_2_CO_3_ (1 mmol), and 0.5 mL of H_2_O in 4.5 mL of DMSO and 80°C*.

**Scheme 1:**
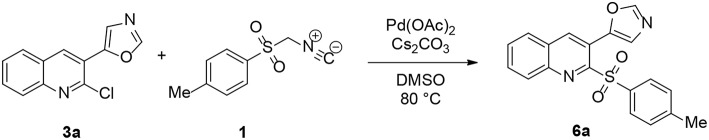
The reaction of TosMIC with **3a**.

The scope of the reaction was explored using tosylmethyl isocyanide (TosMIC) **1** as another isocyanide source. Surprisingly, the reaction of **3a** with TosMIC under the optimized conditions afforded 5-(2-tosylquinolin-3-yl)oxazole (**6a**) (Scheme 1).

Although the reaction proceeded well without a palladium source, the presence of base is crucial. Among the bases Cs_2_CO_3_, K_2_CO_3_, NaOAc, *t*-BuOK, and DABCO, Cs_2_CO_3_ gave the best results.

**Scheme 2:**
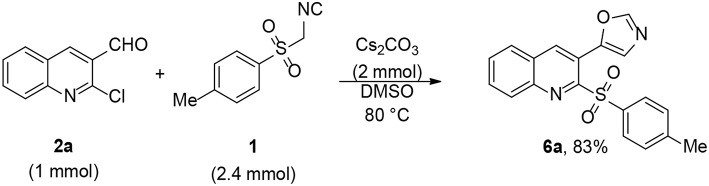
One-pot synthesis of 5-(6-methyl-2-tosylquinolin-3-yl)oxazole **6a**.

Encouraged by the tosylation results with TosMIC, we explored extending the reaction to tandem oxazole formation as well as tosylation of 2-chloroquinoline-3-carbaldehyde. Subjecting 2-chloroquinoline-3-carbaldehyde and TosMIC to the standard reaction conditions yielded 5-(2-tosylquinolin-3-yl)oxazole (**6a**) in 83% yield after 8 h (Scheme 2). Notably, sulfones are present in different bioactive compounds (Metzner and Thuillier, 1994; Fang et al., 2016); however, well-known sulfonylating agents include sulfonyl halides (Tocco et al., 2013; Zhang et al., 2015), sulfonyl hydrazides (Yuan et al., 2018; Zhang et al., 2018), and sodium sulfinate (Sun et al., 2017; Smith et al., 2018). A few studies used TosMIC as a sulfonyl precursor (Liu et al., 2014; Phanindrudu et al., 2016; Kadari et al., 2017). Furthermore, Bounar et al. reacted tosylmethyl isocyanide (TosMIC) with propargylic alcohols in the presence of silver acetate to efficiently yield (*E*)-vinyl sulfones (Bounar et al., 2015); this is the only study in which TosMIC plays a dual role as both an amide and a sulfonyl source.

**Figure 2:**
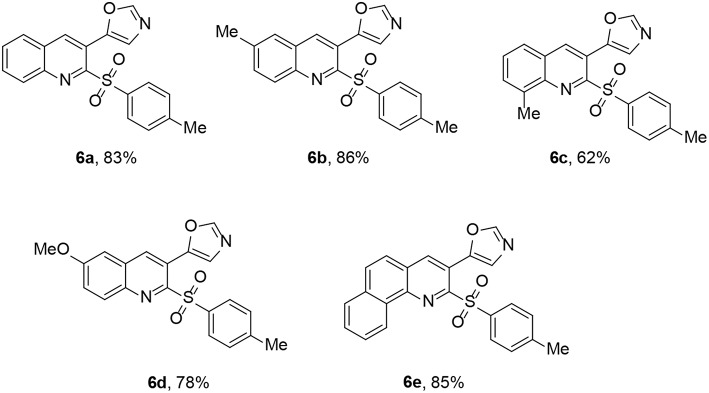
5-(2-Tosylquinolin-3-yl)oxazole **6**.

The above cascade oxazole formation and sulfonylation strategy could be extended to other 2-chloroquinoline-3-carbaldehyde derivatives (Figure 2). A methyl group was tolerated on positions 6 and 8 of **2** to afford **6b** and **6c**, respectively, in 82 and 62% yields. Furthermore, quinoline **2d** reacted with TosMIC, affording **6d** in good yield. Product **6e**, existing an alternative decoration of the quinoline ring, was obtained in 85% yield.

Our proposed mechanism for the tosylation of quinoline involved *in situ* Ts^−^ generation by decomposition of *p*-toluenesulfonylmethyl-isocyanide **1** in the presence of base with subsequent aromatic nucleophilic substitution to form 2-sulfonyl quinoline **6**. Although application of TosMIC as a sulfonyl source was reported by Liu et al. for synthesizing sulfonyl benzoheteroles, the sulfonation mechanism involved aliphatic nucleophilic substitution (Liu et al., 2014).

## Conclusion

In summary, we have developed a synthesis of 5-(2-chloroquinolin-3-yl)oxazole via a van Leusen procedure from 2-chloroquinoline-3-carbaldehydes and TosMIC, which were efficiently subjected to Pd-catalyzed amidation with isocyanides to form 3-(oxazol-5-yl)quinoline-2-carboxamides. The synthesis of 5-(2-tosylquinolin-3-yl)oxazole via a Cs_2_CO_3_-mediated domino process starting from 2-chloroquinoline-3-carbaldehydes with TosMIC was also demonstrated.

## Author Contributions

ZM, ZT, and SF synthesized all of the compounds with the help of ZY. MS supervised this work and wrote the paper with the help of ZY.

### Conflict of Interest Statement

The authors declare that the research was conducted in the absence of any commercial or financial relationships that could be construed as a potential conflict of interest.

## Supplementary Material

Supplementary Data Sheet 1 Isocyanide reactions toward the synthesis of 3-(Oxazol-5-yl)quinoline-2-carboxamides and 5-(2-Tosylquinolin-3-yl)oxazole.
