# Supplementary material for: Isocyanide Reactions Toward the Synthesis of 3-(Oxazol-5-yl)Quinoline-2-Carboxamides and 5-(2-Tosylquinolin-3-yl)Oxazole
Source: Front Chem. 2019 Jun 14;7:433. doi: 10.3389/fchem.2019.00433 (PMC6587330; doi:10.3389/fchem.2019.00433)
Supplement: Supplementary Data Sheet 1 — Isocyanide reactions toward the synthesis of 3-(Oxazol-5-yl)quinoline-2-carboxamides and 5-(2-Tosylquinolin-3-yl)oxazole. [file Data_Sheet_1.PDF]

NMR
Sample code:Y-Z-C (Dr.Shiri)

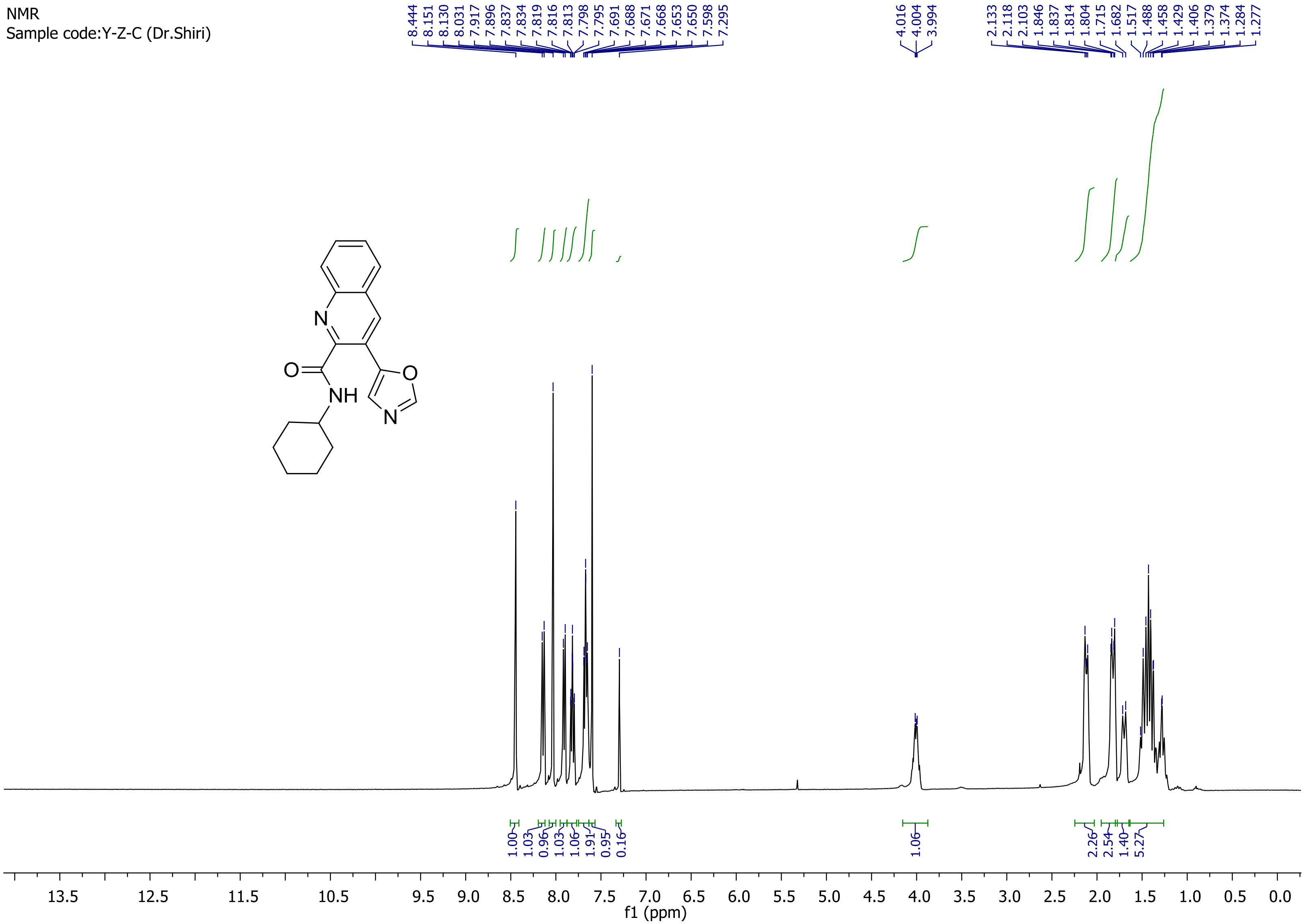

NMR
Sample code:Y-Z-C (Dr.Shiri)

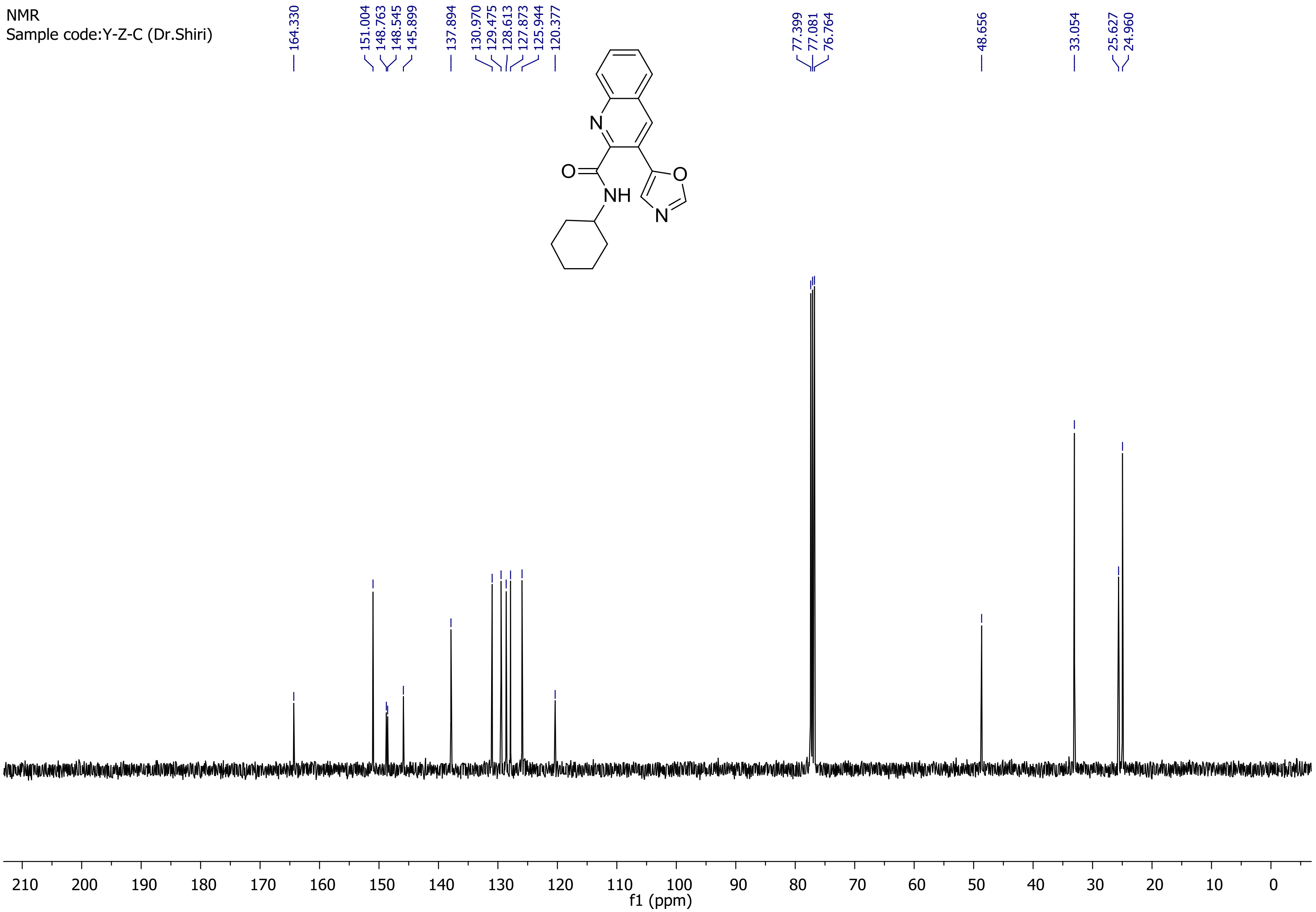

Al-zahra UN
Sample code: NSY2T (Dr. Shiri)

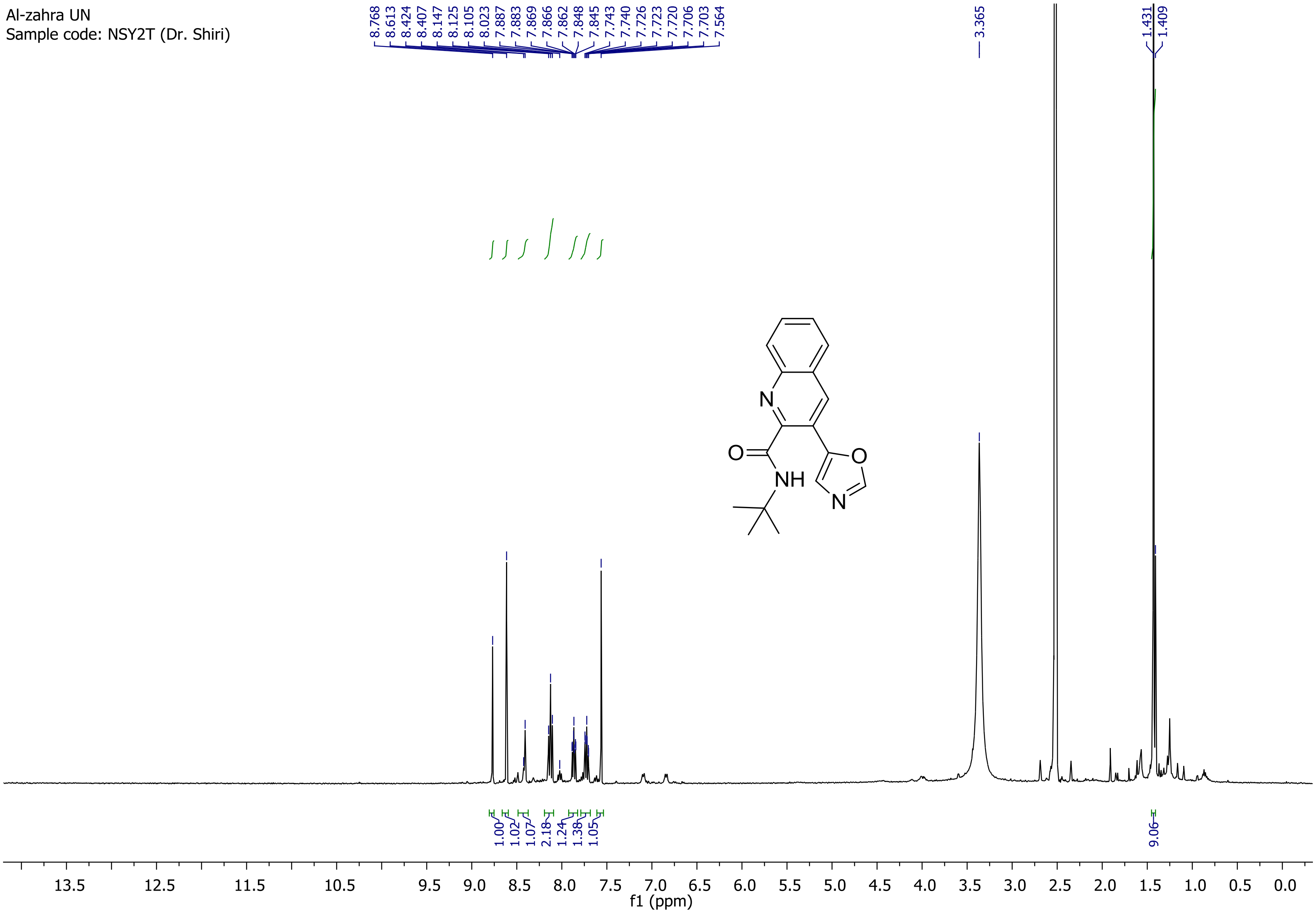

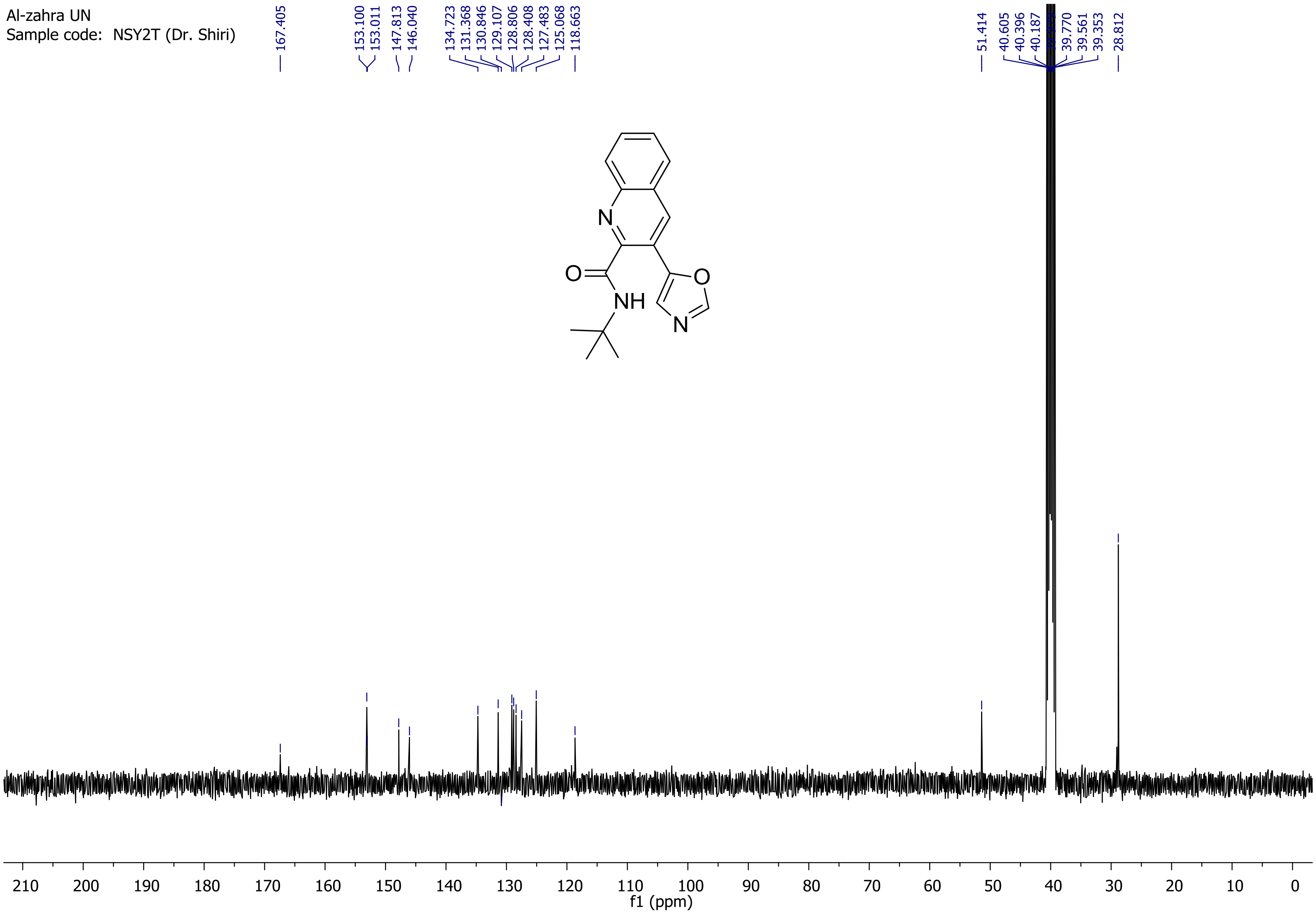
Al-zahra UN
Sample code: NSY2T (Dr. Shiri)
167.405
153.100
153.011
147.813
146.040
134.723
131.368
130.846
129.107
128.806
128.408
127.483
125.068
118.663
51.414
40.605
40.396
40.187
39.770
39.561
39.353
28.812
N
O
NH
O
N
f1 (ppm)
210
200
190
180
170
160
150
140
130
120
110
100
90
80
70
60
50
40
30
20
10
0

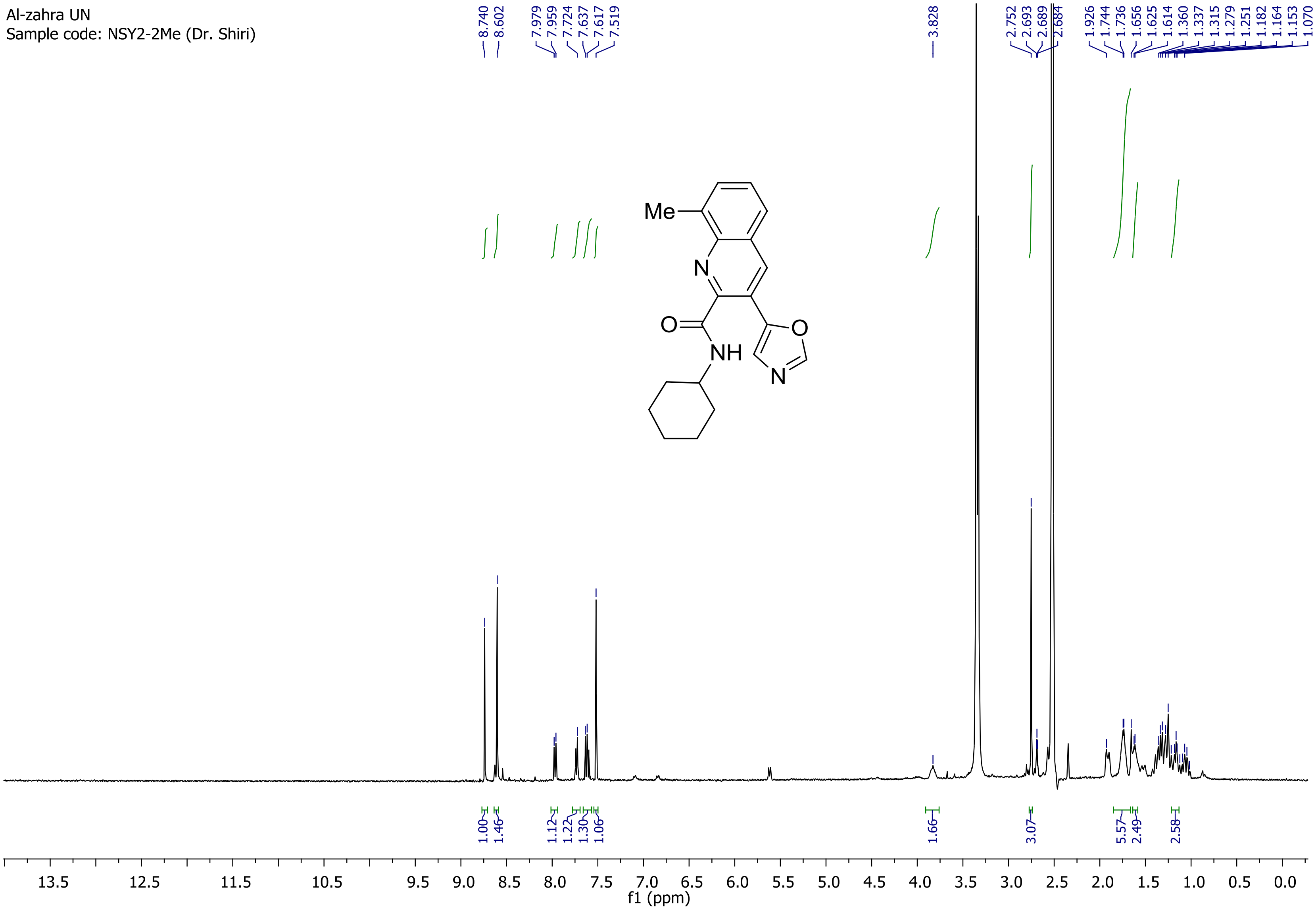
Al-zahra UN
Sample code: NSY2-2Me (Dr. Shiri)
8.740
8.602
7.979
7.959
7.724
7.637
7.617
7.519
3.828
2.752
2.693
2.689
2.684
1.926
1.744
1.736
1.656
1.625
1.614
1.360
1.337
1.315
1.279
1.251
1.182
1.164
1.153
1.070
Me
N
O
NH
O
N
1.00
1.46
1.12
1.22
1.30
1.06
1.66
3.07
5.57
2.49
2.58
13.5
12.5
11.5
10.5
9.5
9.0
8.5
8.0
7.5
7.0
6.5
6.0
5.5
5.0
4.5
4.0
3.5
3.0
2.5
2.0
1.5
1.0
0.5
0.0
f1 (ppm)

Al-zahra UN
Sample code: NSY2-2Me (Dr. Shiri)

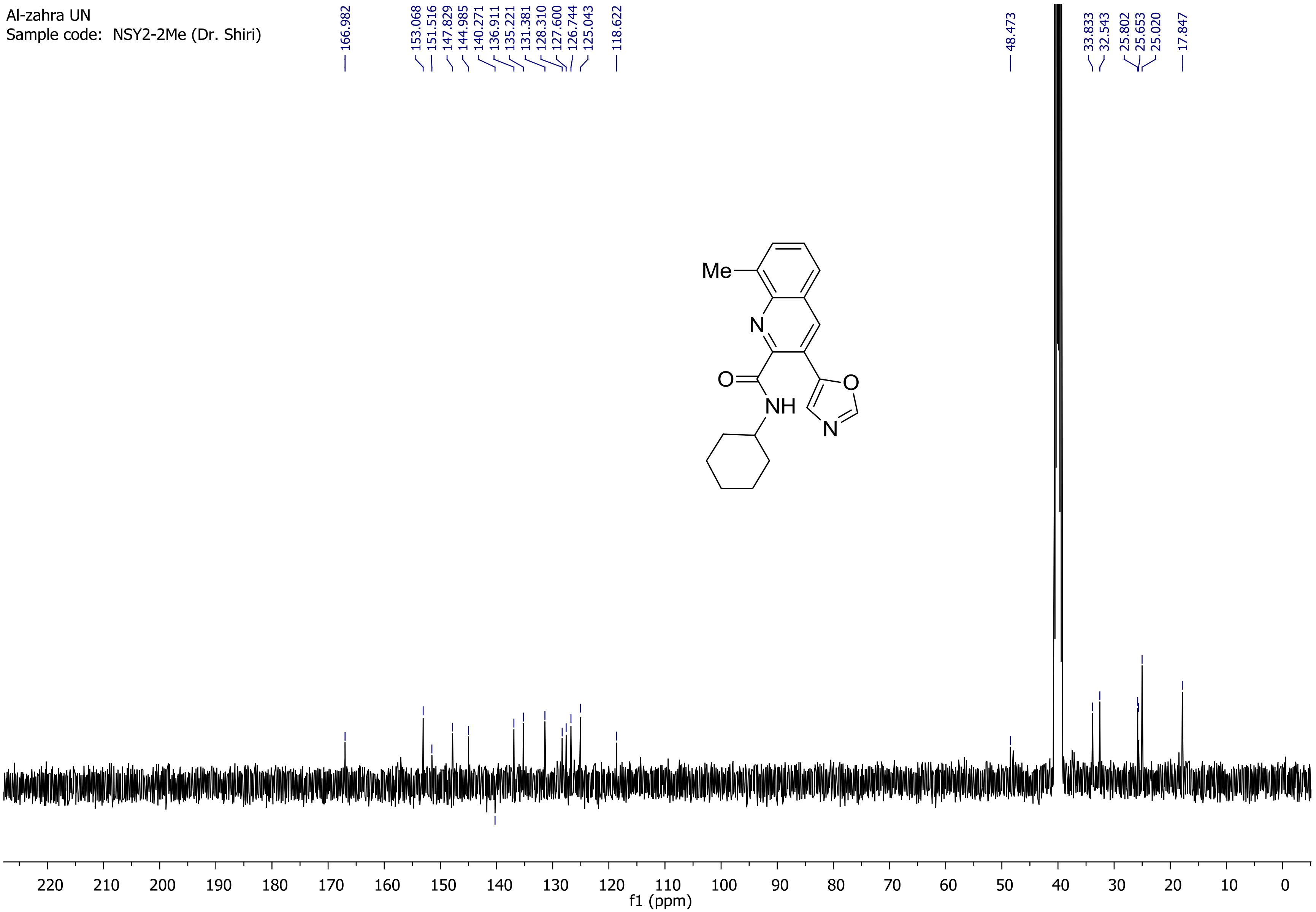

NMR
Sample code: Y-Cl

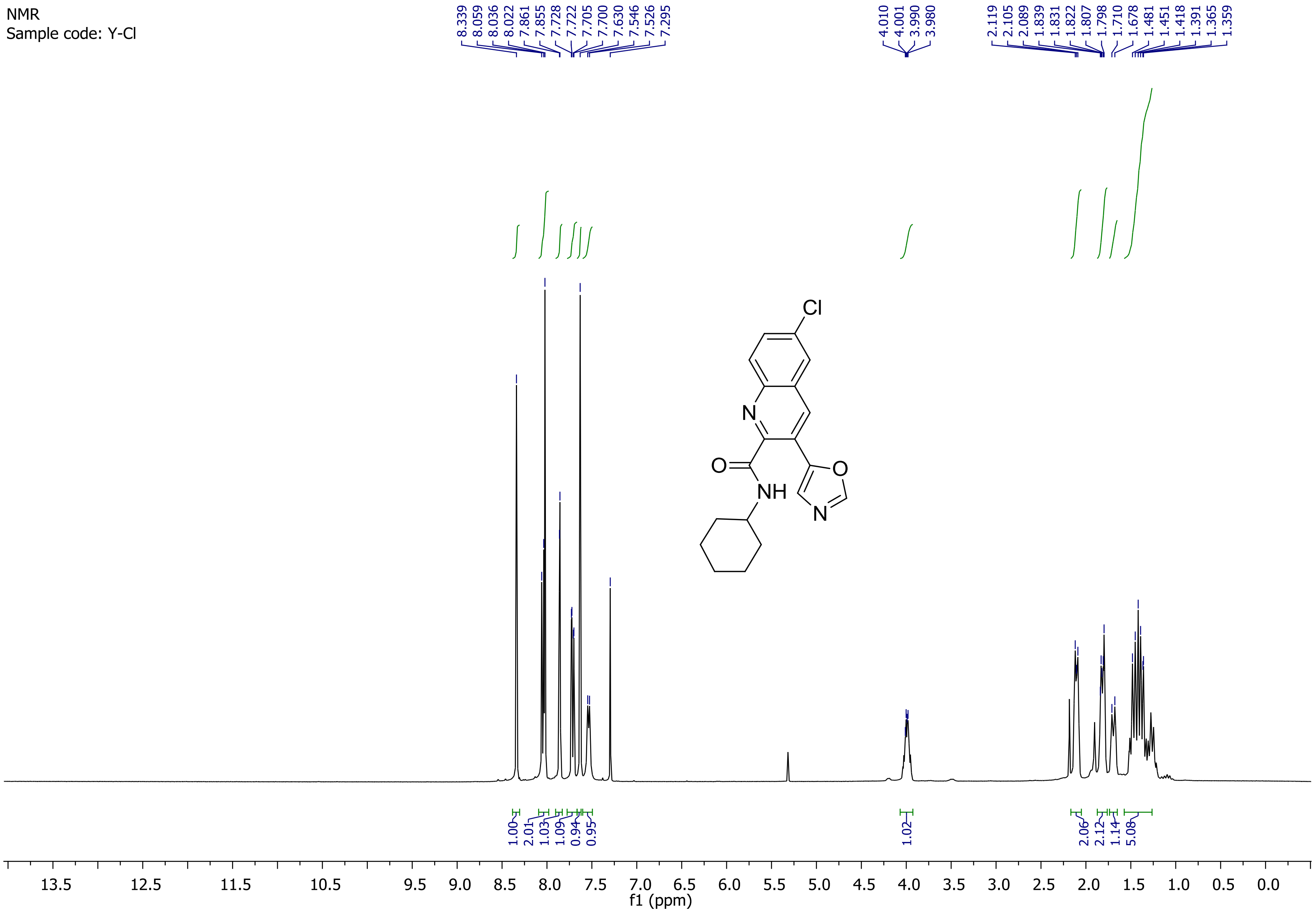

NMR
Sample code: Y-Cl

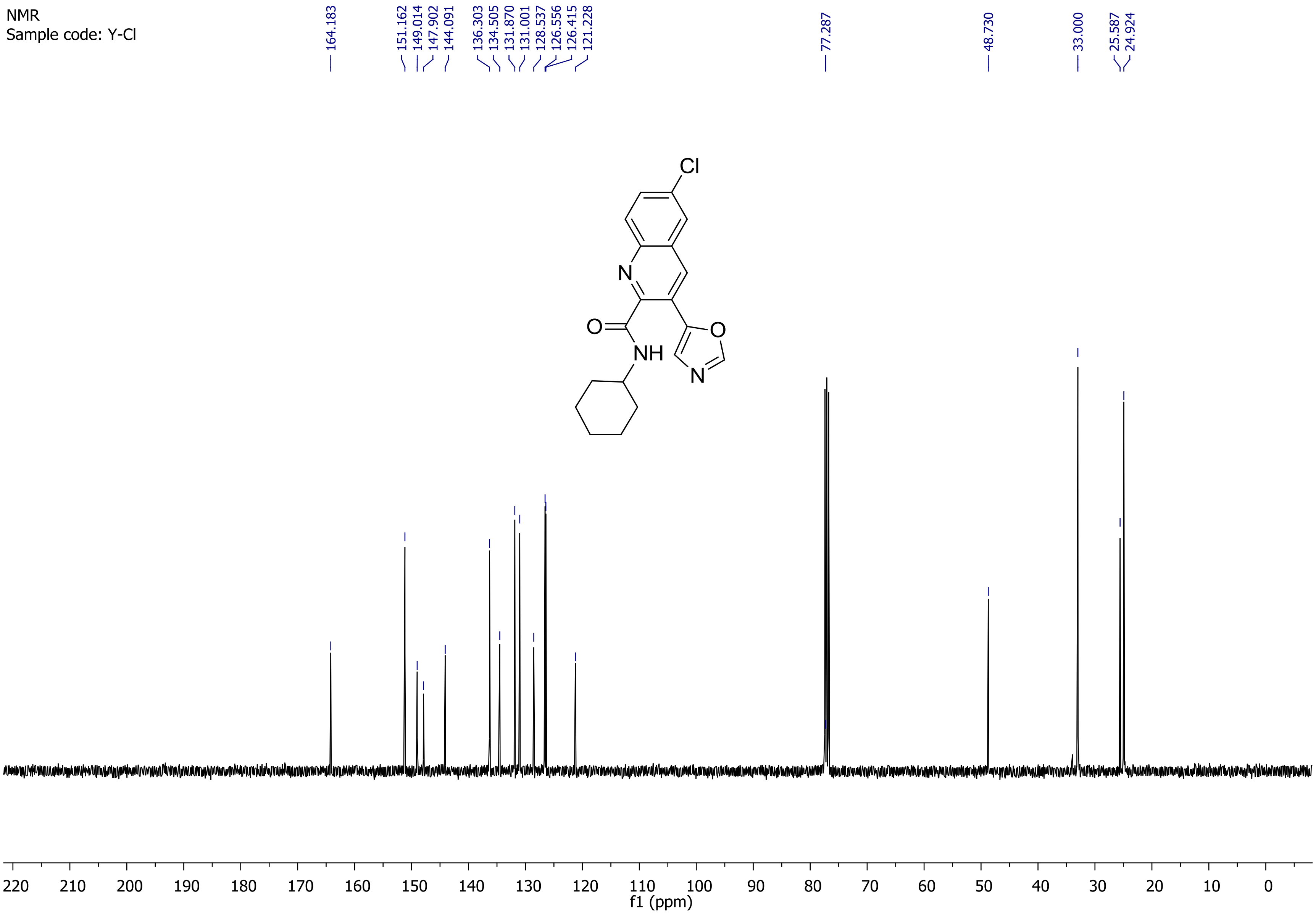

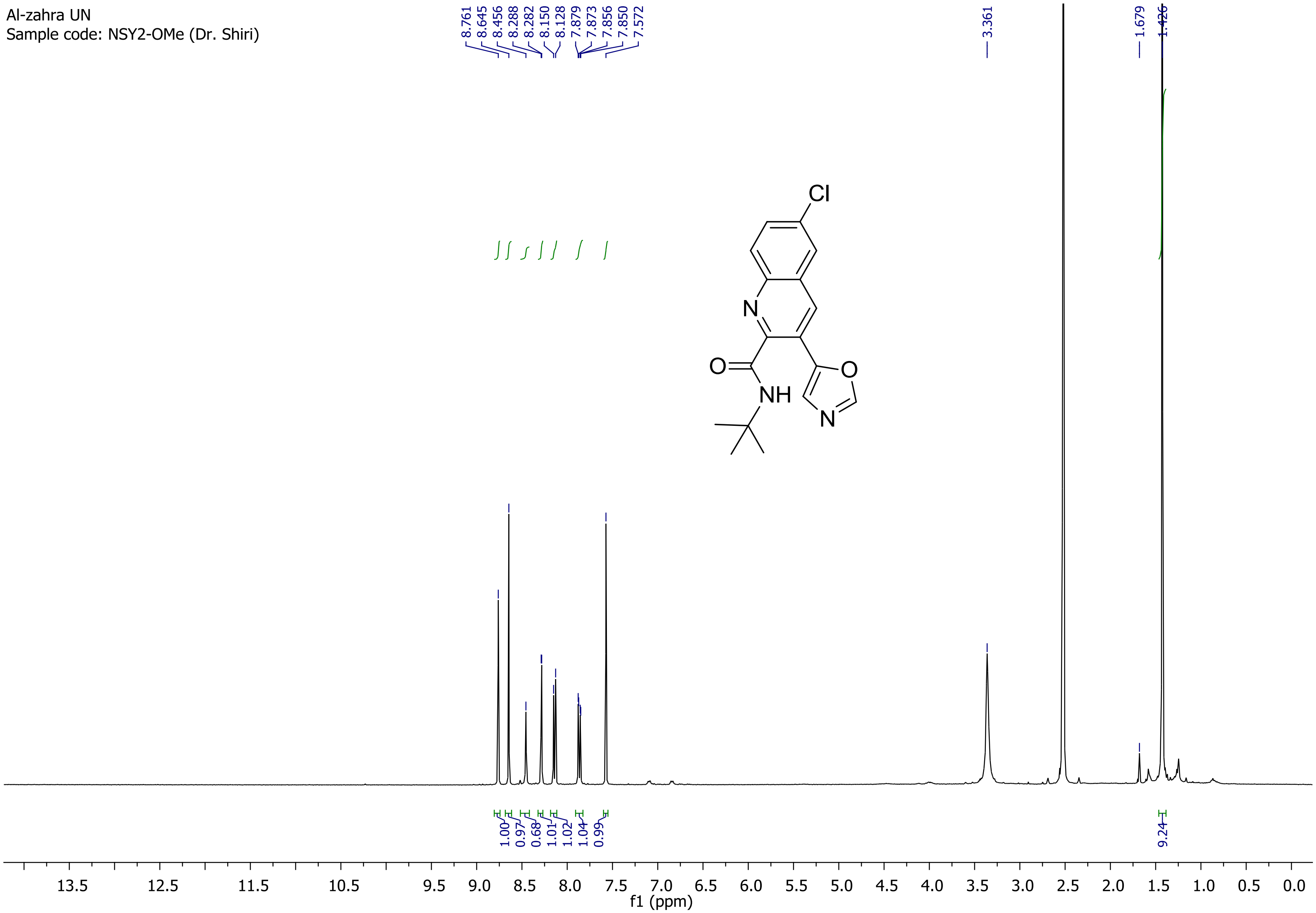
Al-zahra UN
Sample code: NSY2-OMe (Dr. Shiri)
8.761
8.645
8.456
8.288
8.282
8.150
8.128
7.879
7.873
7.856
7.850
7.572
3.361
1.679
1.426
Cl
N
O
NH
O
N
1.00
0.97
0.68
1.01
1.02
1.04
0.99
9.24
13.5
12.5
11.5
10.5
9.5
9.0
8.5
8.0
7.5
7.0
6.5
6.0
5.5
5.0
4.5
4.0
3.5
3.0
2.5
2.0
1.5
1.0
0.5
0.0
f1 (ppm)

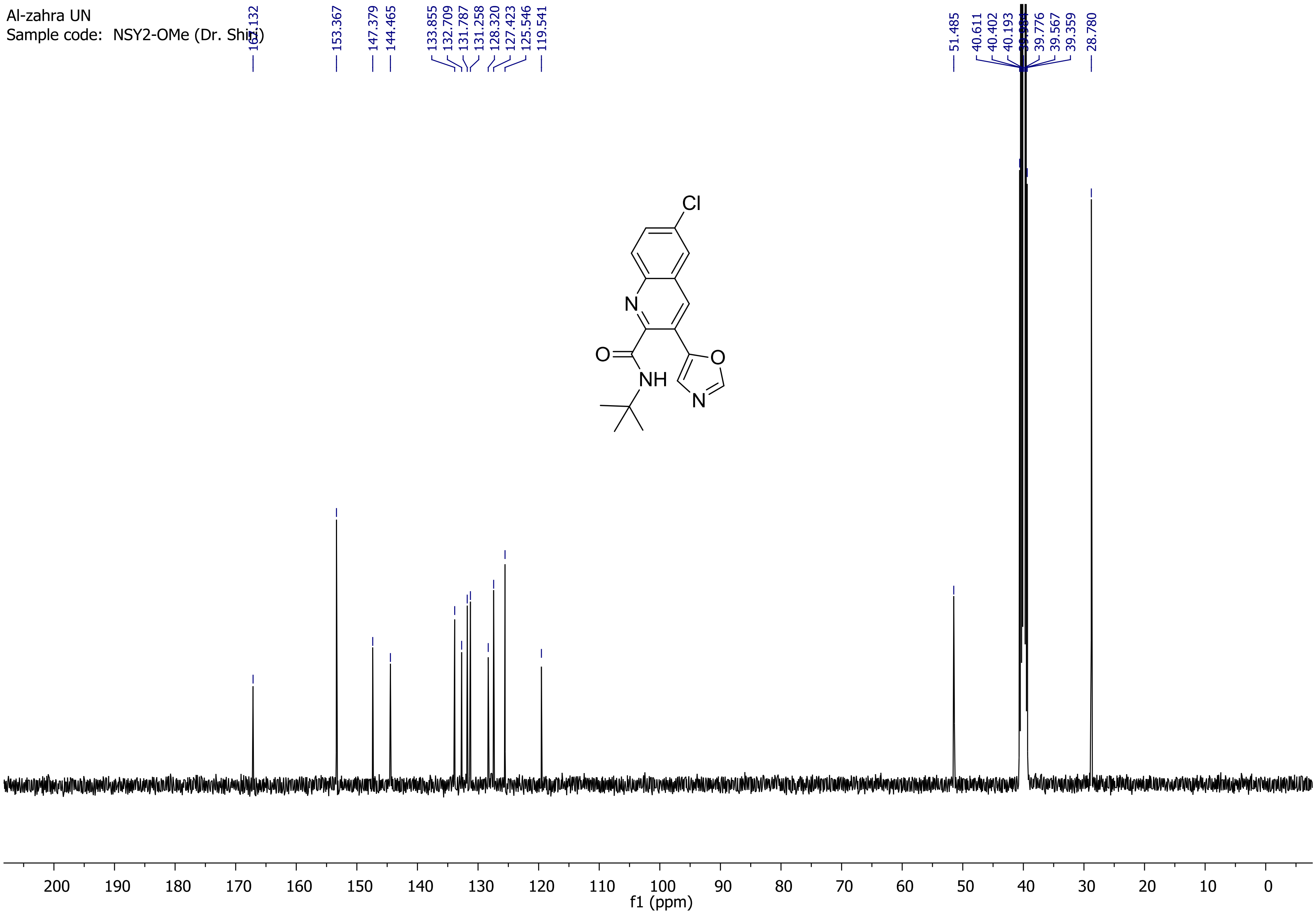
Al-zahra UN
Sample code: NSY2-OMe (Dr. Shiri)
167.132
153.367
147.379
144.465
133.855
132.709
131.787
131.258
128.320
127.423
125.546
119.541
51.485
40.611
40.402
40.193
39.984
39.776
39.567
39.359
28.780
Cl
N
O
NH
O
N
200
190
180
170
160
150
140
130
120
110
100
90
80
70
60
50
40
30
20
10
0
f1 (ppm)

Al-zahra UN
Sample code: NSY2T-Cl (Dr. Shiri)

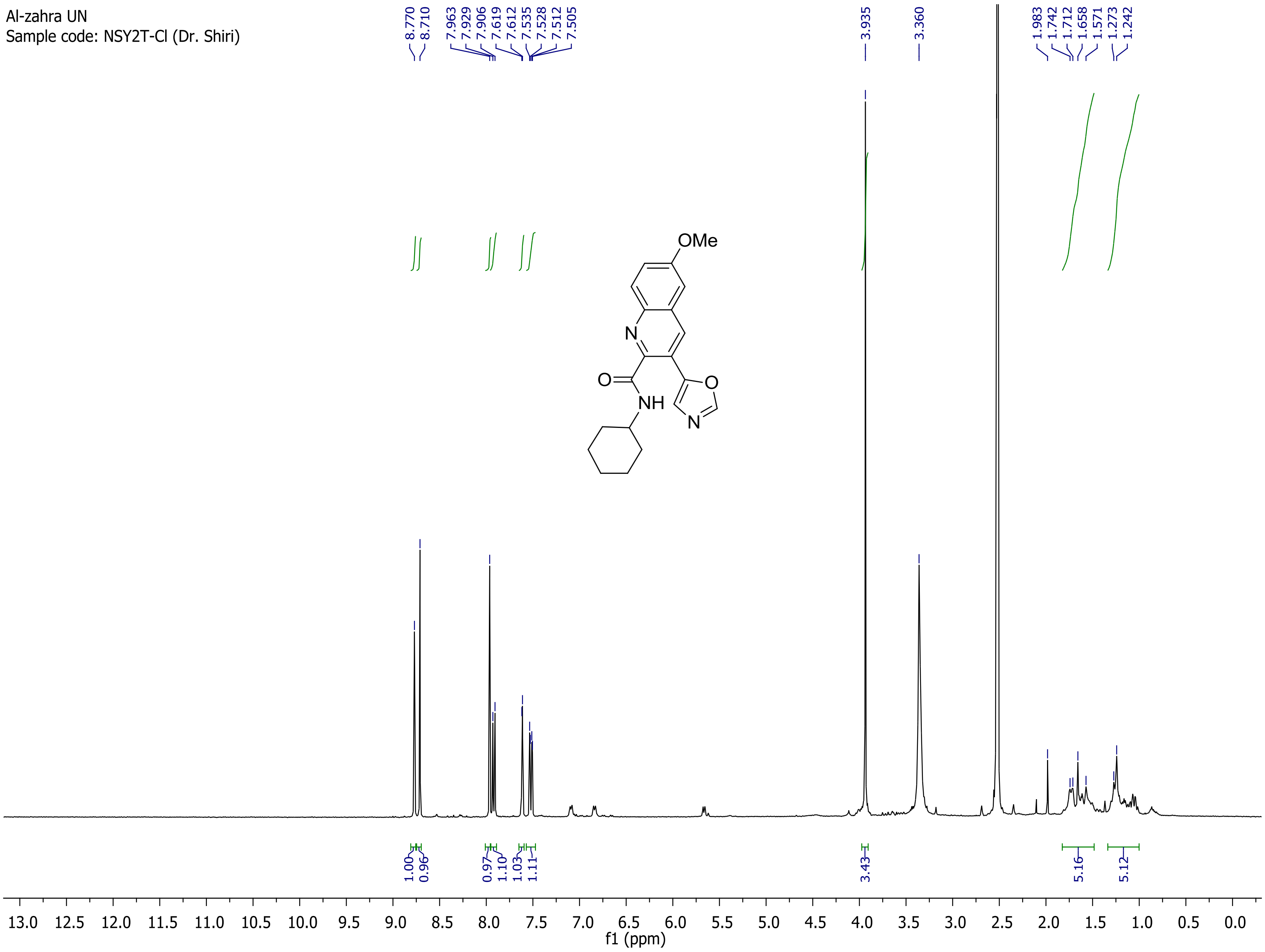

Al-zahra UN
Sample code: NSY2T-Cl (Dr. Shiri)

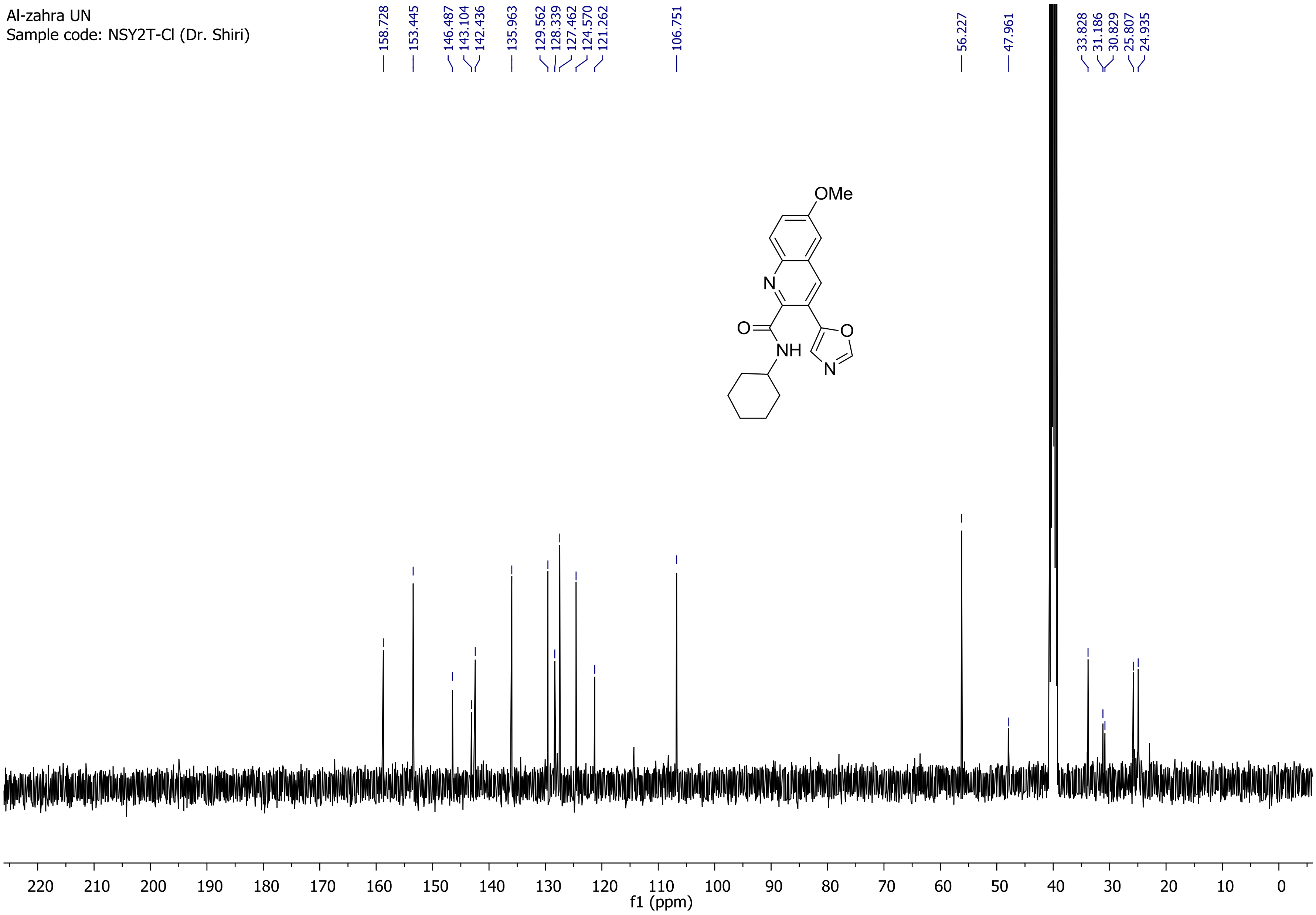

NMR
Sample code: Y-B

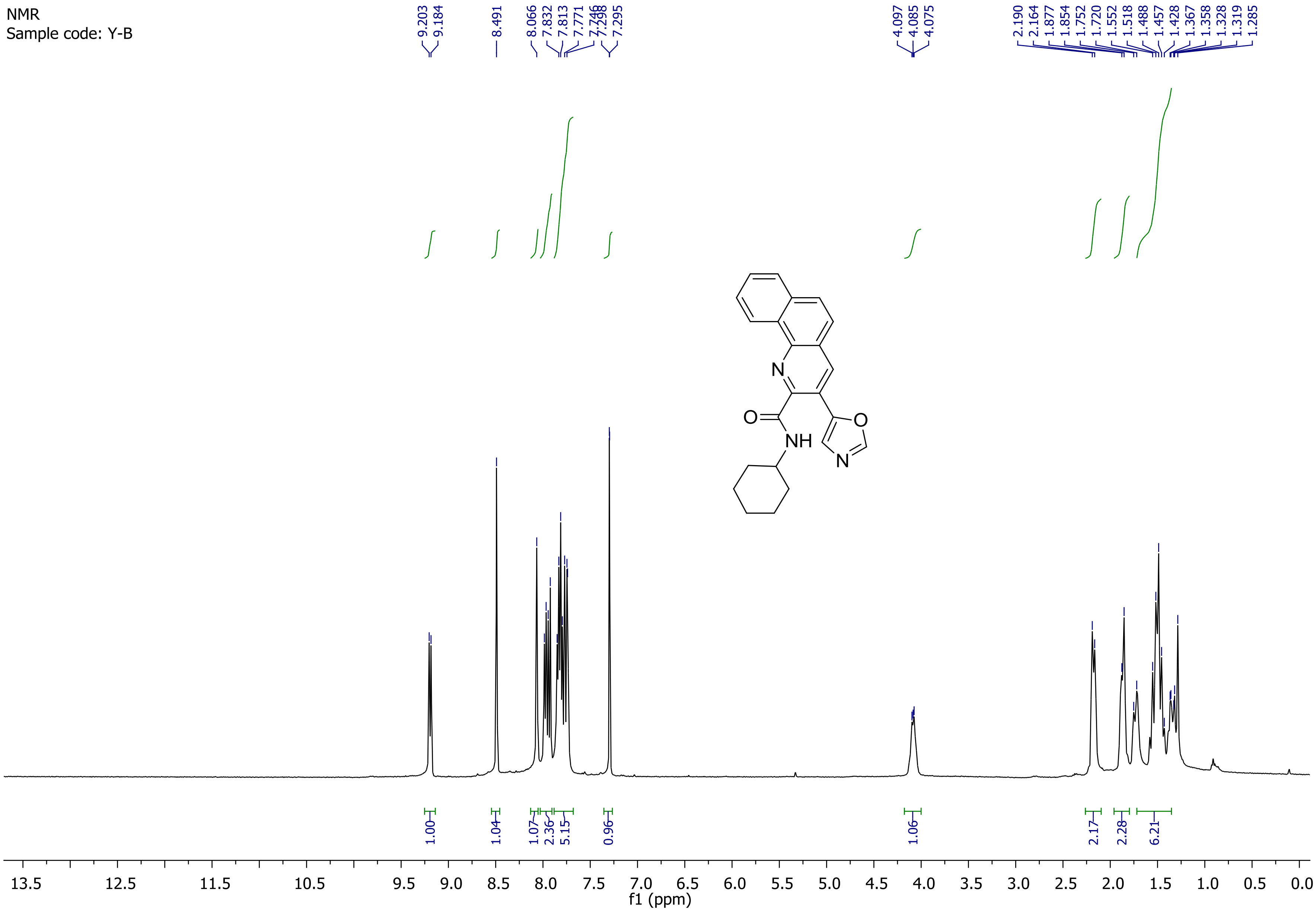

NMR
Sample code: Y-B

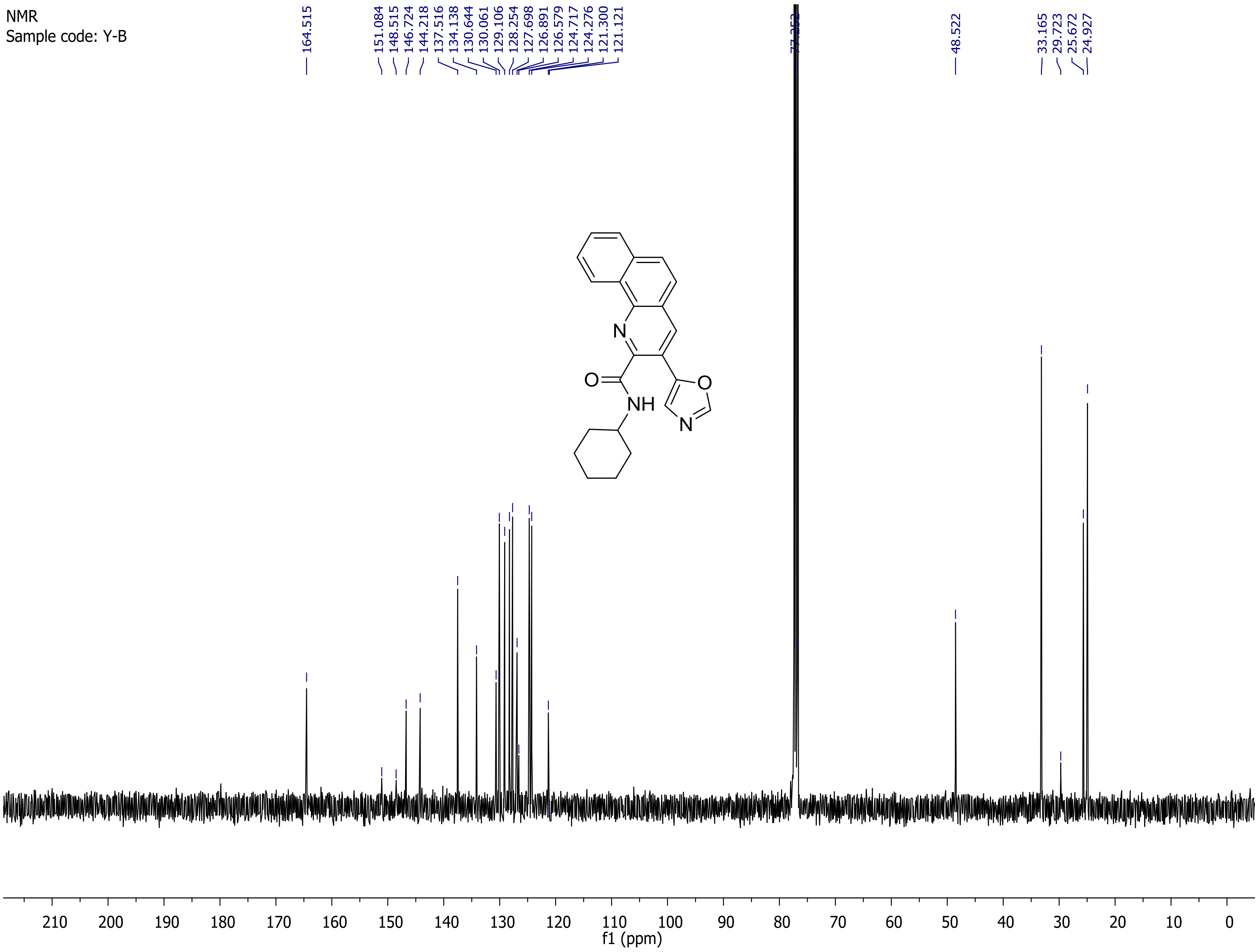

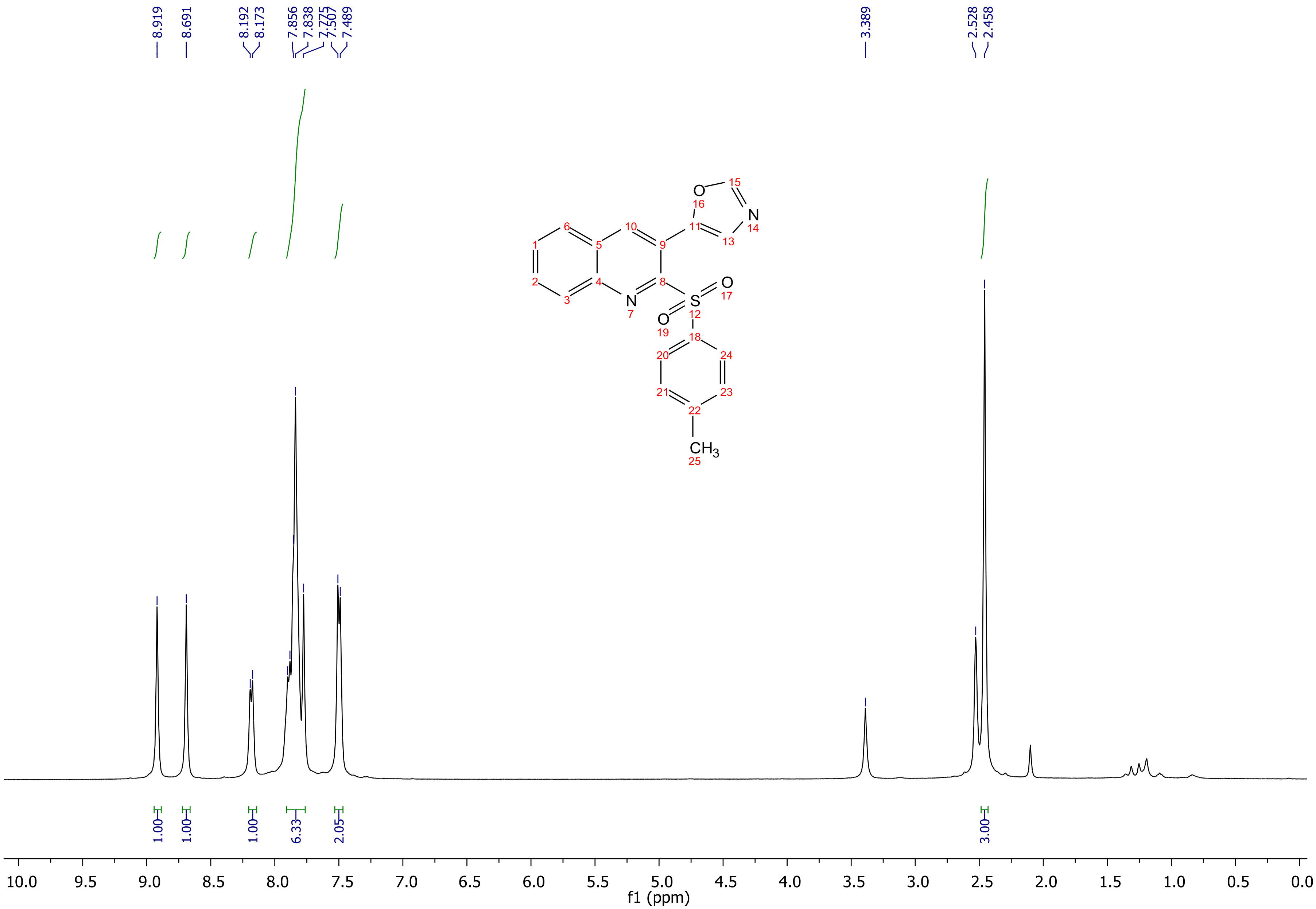

8.919
8.691
8.192
8.173
7.856
7.838
7.775
7.507
7.489
3.389
2.528
2.458
1.00
1.00
1.00
6.33
2.05
3.00
10.0 9.5 9.0 8.5 8.0 7.5 7.0 6.5 6.0 5.5 5.0 4.5 4.0 3.5 3.0 2.5 2.0 1.5 1.0 0.5 0.0
f1 (ppm)

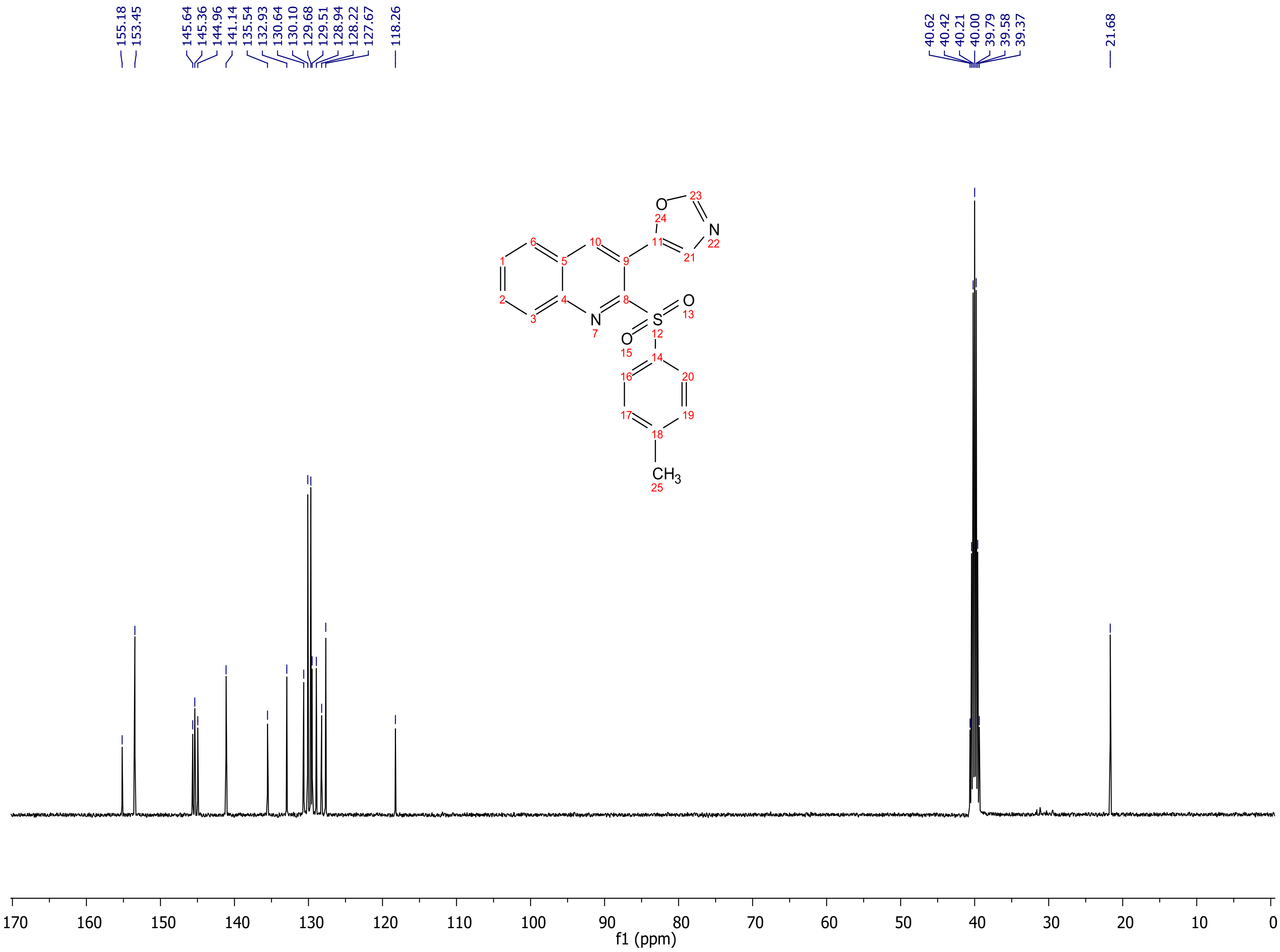

155.18
153.45
145.64
145.36
144.96
141.14
135.54
132.93
130.64
130.10
129.68
129.51
128.94
128.22
127.67
118.26
40.62
40.42
40.21
40.00
39.79
39.58
39.37
21.68
O
N
S
CH3
170 160 150 140 130 120 110 100 90 80 70 60 50 40 30 20 10 0
f1 (ppm)

Al-zahra UN
Sample Code:YZ2MeS (Dr.Shiri)

	Parameter	Value
1	Data File Name	D:/ Zahra/ My Articles/ Article-Mohammadpour+Fazelzadeh/ NMR/ new/ Al-zahra UN/ 760/ fid
2	Title	Al-zahra UN
3	Comment	Sample Code:YZ2MeS (Dr.Shiri)
4	Origin	Bruker BioSpin GmbH
5	Owner	Administrator
6	Site	
7	Spectrometer	spect
8	Author	
9	Solvent	DMSO
10	Temperature	293.3
11	Pulse Sequence	zg30
12	Number of Scans	20
13	Receiver Gain	256
14	Relaxation Delay	4.0000
15	Pulse Width	14.0000
16	Acquisition Time	4.0894
17	Acquisition Date	2018-05-30T08:51:31
18	Modification Date	2018-05-30T08:51:34
19	Spectrometer Frequency	400.22
20	Spectral Width	8012.8
21	Lowest Frequency	-404.4
22	Nucleus	1H
23	Acquired Size	32768
24	Spectral Size	65536

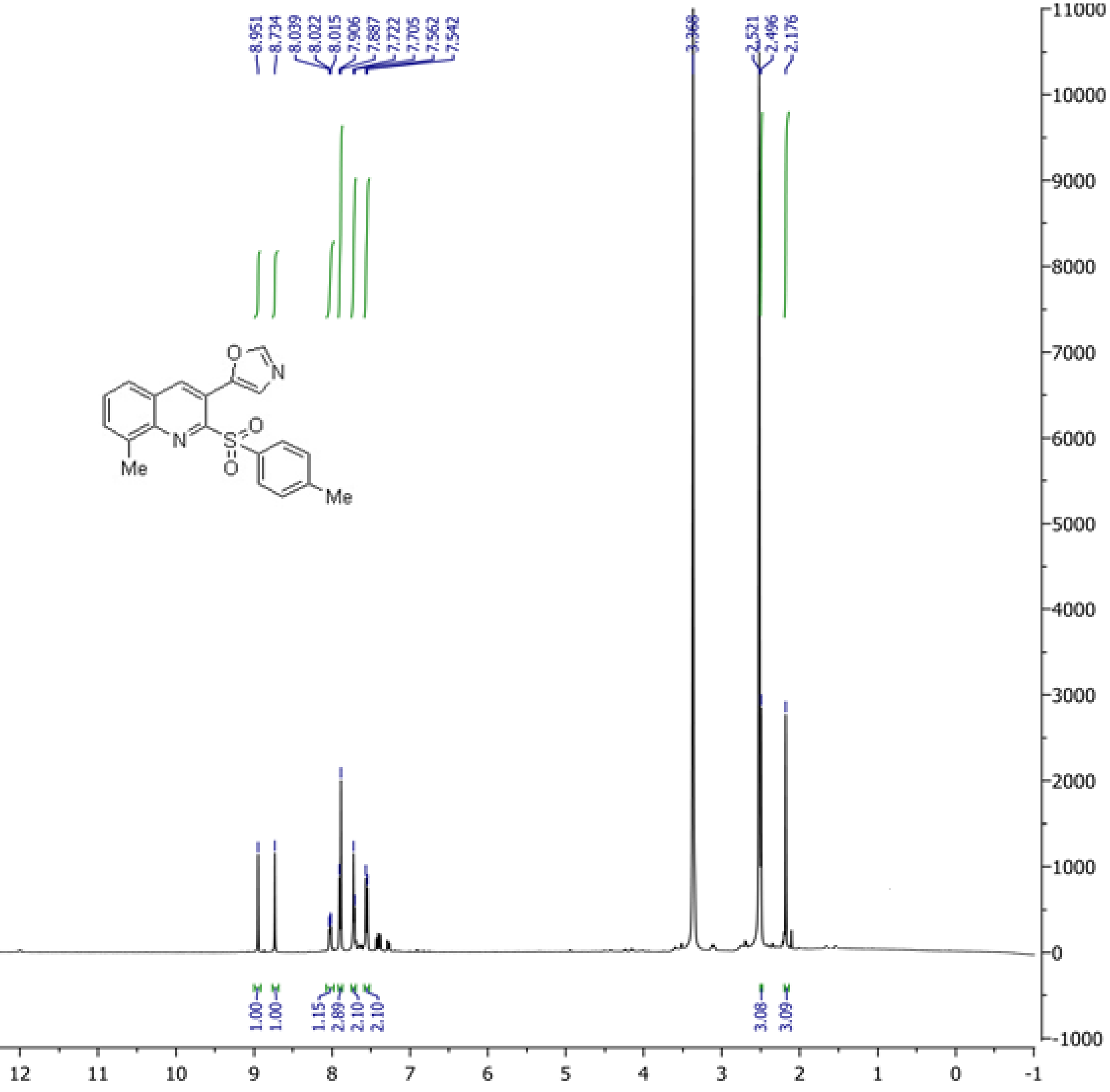

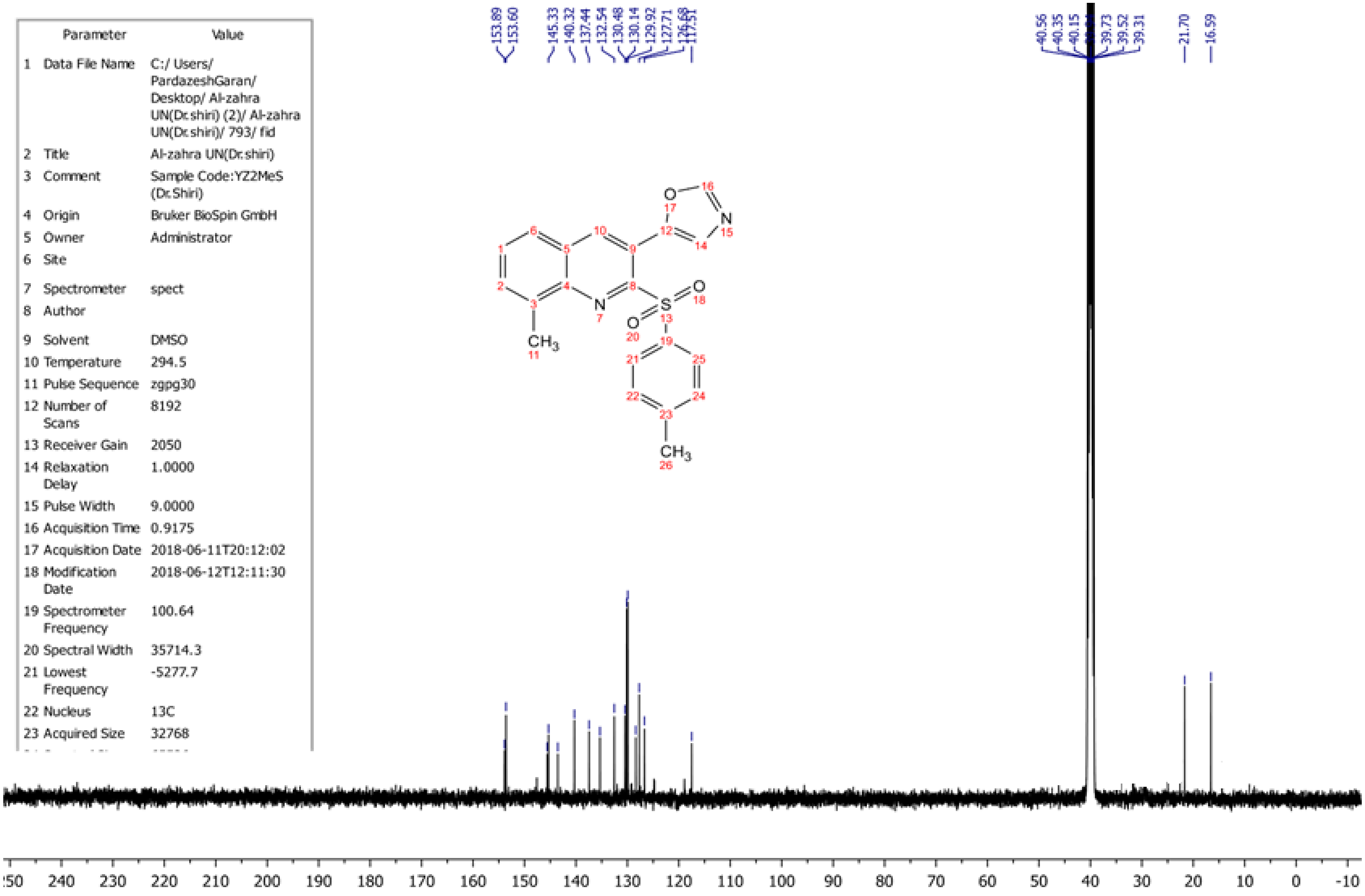

	Parameter	Value
1	Data File Name	C:/ Users/ PardazeshGaran/ Desktop/ Al-zahra UN(Dr.shiri) (2)/ Al-zahra UN(Dr.shiri)/ 793/ fid
2	Title	Al-zahra UN(Dr.shiri)
3	Comment	Sample Code:YZ2MeS (Dr.Shiri)
4	Origin	Bruker BioSpin GmbH
5	Owner	Administrator
6	Site	
7	Spectrometer	spect
8	Author	
9	Solvent	DMSO
10	Temperature	294.5
11	Pulse Sequence	zgpg30
12	Number of Scans	8192
13	Receiver Gain	2050
14	Relaxation Delay	1.0000
15	Pulse Width	9.0000
16	Acquisition Time	0.9175
17	Acquisition Date	2018-06-11T20:12:02
18	Modification Date	2018-06-12T12:11:30
19	Spectrometer Frequency	100.64
20	Spectral Width	35714.3
21	Lowest Frequency	-5277.7
22	Nucleus	13C
23	Acquired Size	32768
[illegible]	[illegible]	[illegible]

Al-zahra UN
Sample Code:yzs4Me (Dr.Shiri)

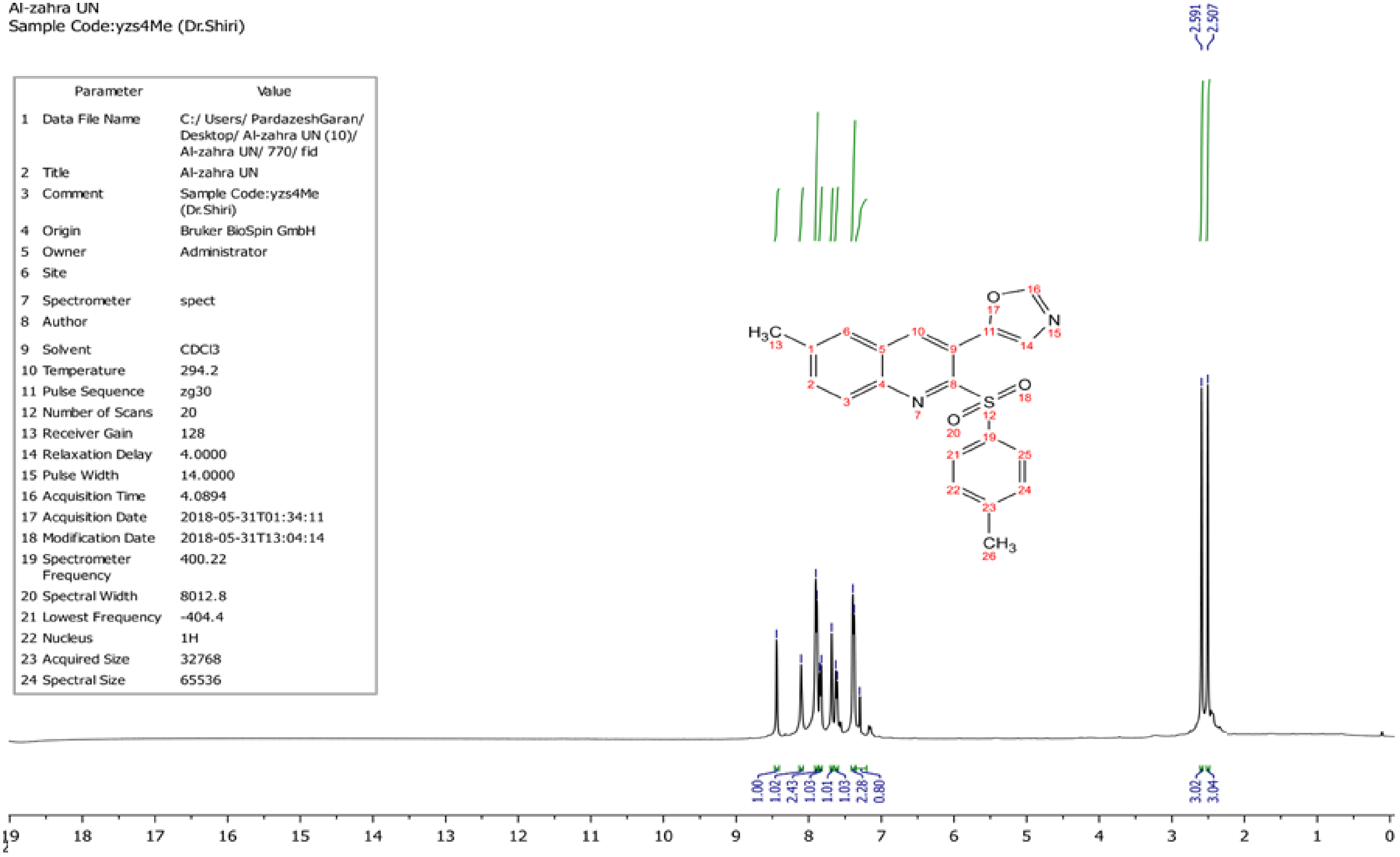

	Parameter	Value
1	Data File Name	C:/ Users/ PardazeshGaran/ Desktop/ Al-zahra UN (10)/ Al-zahra UN/ 770/ fid
2	Title	Al-zahra UN
3	Comment	Sample Code:yzs4Me (Dr.Shiri)
4	Origin	Bruker BioSpin GmbH
5	Owner	Administrator
6	Site	
7	Spectrometer	spect
8	Author	
9	Solvent	CDCl3
10	Temperature	294.2
11	Pulse Sequence	zg30
12	Number of Scans	20
13	Receiver Gain	128
14	Relaxation Delay	4.0000
15	Pulse Width	14.0000
16	Acquisition Time	4.0894
17	Acquisition Date	2018-05-31T01:34:11
18	Modification Date	2018-05-31T13:04:14
19	Spectrometer Frequency	400.22
20	Spectral Width	8012.8
21	Lowest Frequency	-404.4
22	Nucleus	1H
23	Acquired Size	32768
24	Spectral Size	65536

	Parameter	Value
1	Data File Name	C:/ Users/ PardazeshGaran/ Desktop/ NMR-NEW/ 258/ fid
2	Title	Dr.shiri
3	Comment	Y-Z-S-O-2<br>13C{1H} NMR
4	Origin	UXNMR, Bruker Analytische Messtechnik GmbH
5	Owner	root
6	Site	
7	Spectrometer	spect
8	Author	
9	Solvent	DMSO
10	Temperature	300.0
11	Pulse Sequence	zgpg30
12	Number of Scans	1743
13	Receiver Gain	2048
14	Relaxation Delay	2.0000
15	Pulse Width	130.0000
16	Acquisition Time	1.8220
17	Acquisition Date	2014-02-12T10:18:00
18	Modification Date	2014-02-12T11:22:18
19	Spectrometer Frequency	75.47
20	Spectral Width	17985.6
21	Lowest Frequency	-1446.5
22	Nucleus	13C
23	Acquired Size	32768
24	Spectral Size	65536

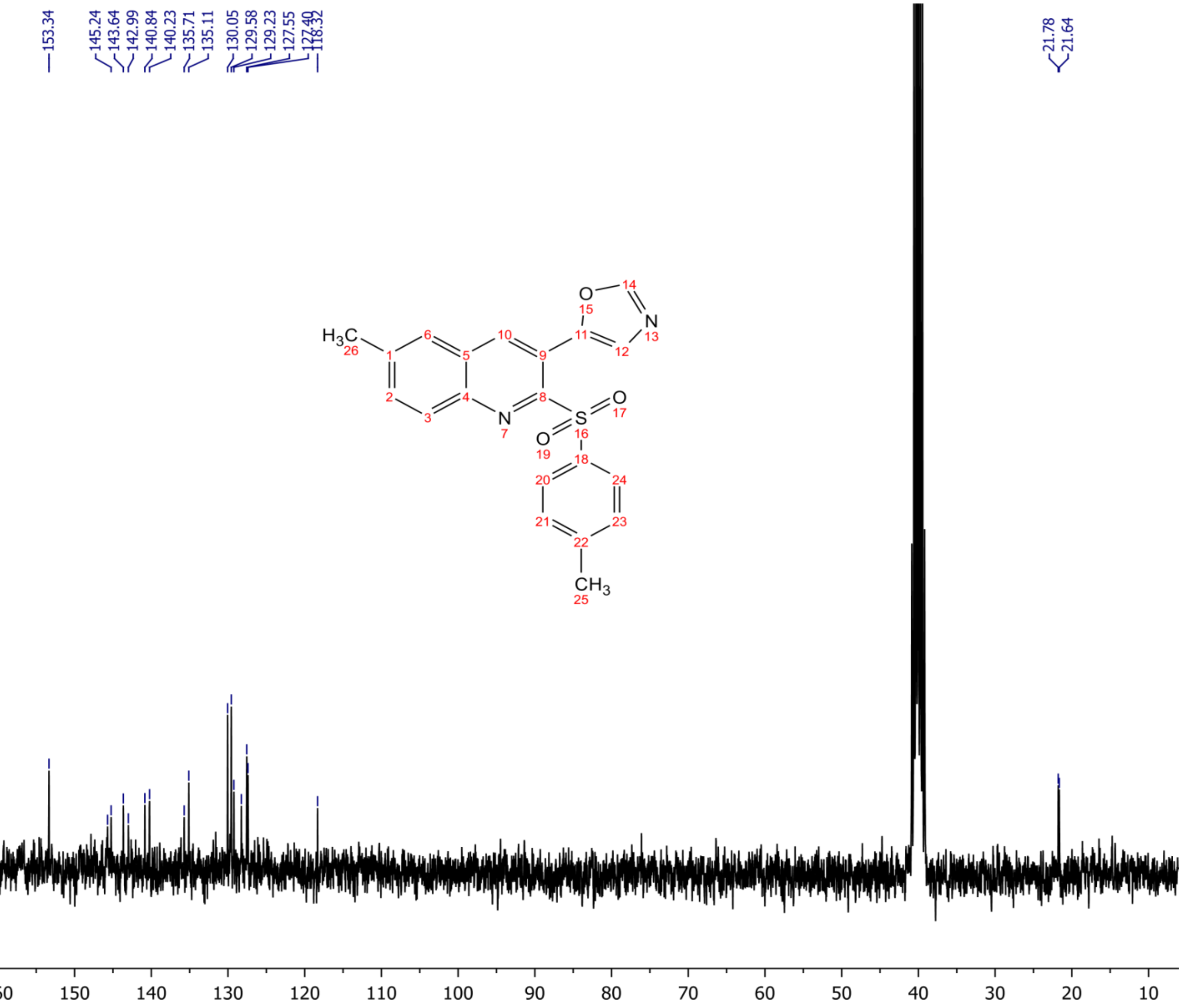

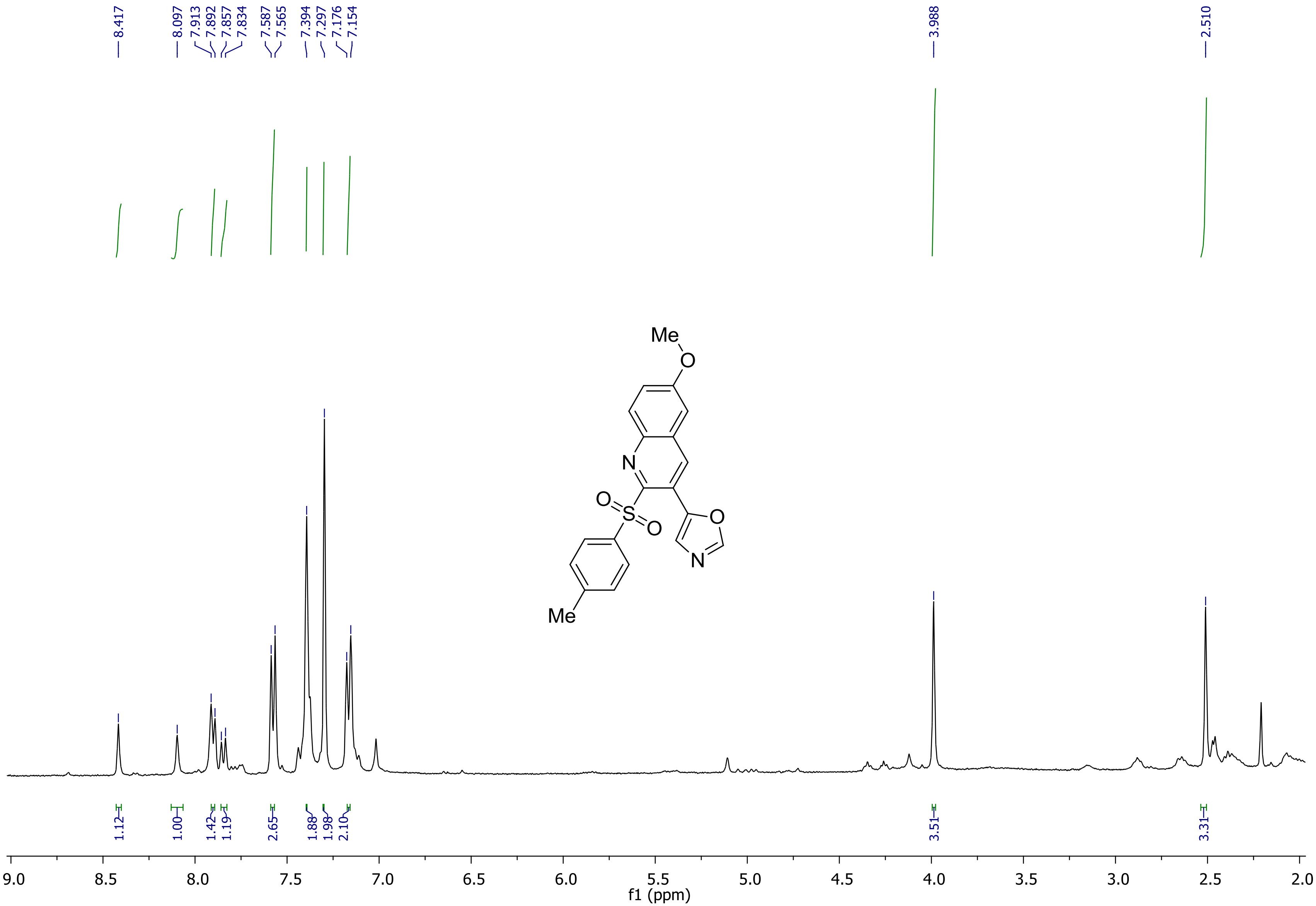

8.417
8.097
7.913
7.892
7.857
7.834
7.587
7.565
7.394
7.297
7.176
7.154
3.988
2.510
Me
O
N
O
S
O
O
N
Me
1.12
1.00
1.42
1.19
2.65
1.88
1.98
2.10
3.51
3.31
9.0
8.5
8.0
7.5
7.0
6.5
6.0
5.5
5.0
4.5
4.0
3.5
3.0
2.5
2.0
f1 (ppm)

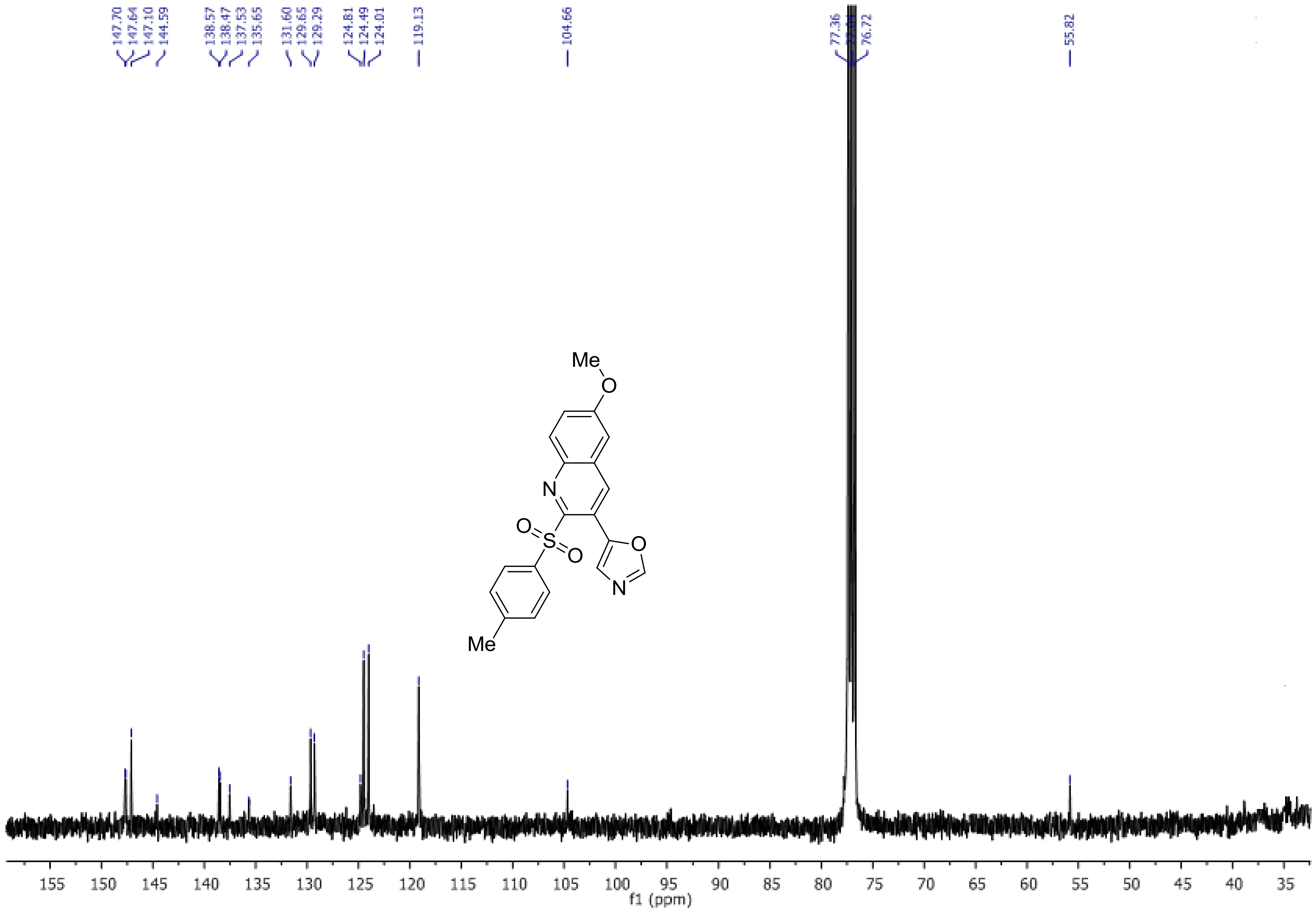

147.70
147.64
147.10
144.59
138.57
138.47
137.53
135.65
131.60
129.65
129.29
124.81
124.49
124.01
119.13
104.66
77.36
76.72
55.82
Me
O
N
O
S
O
O
N
Me
155 150 145 140 135 130 125 120 115 110 105 100 95 90 85 80 75 70 65 60 55 50 45 40 35
f1 (ppm)

	Parameter	Value
1	Data File Name	D:/ Z.M-W/ /2 کار 749/ fid
2	Title	2 کار
3	Comment	Sample code:y-z-as (Dr-shiri)
4	Origin	Bruker BioSpin GmbH
5	Owner	Administrator
6	Site	
7	Spectrometer	spect
8	Author	
9	Solvent	CDCl3
10	Temperature	292.9
11	Pulse Sequence	zg30
12	Number of Scans	20
13	Receiver Gain	144
14	Relaxation Delay	4.0000
15	Pulse Width	14.0000
16	Acquisition Time	4.0894
17	Acquisition Date	2018-05-11T19:57:37
18	Modification Date	2018-05-12T07:27:40
19	Spectrometer Frequency	400.22
20	Spectral Width	8012.8
21	Lowest Frequency	-404.4
22	Nucleus	1H
23	Acquired Size	32768
24	Spectral Size	65536

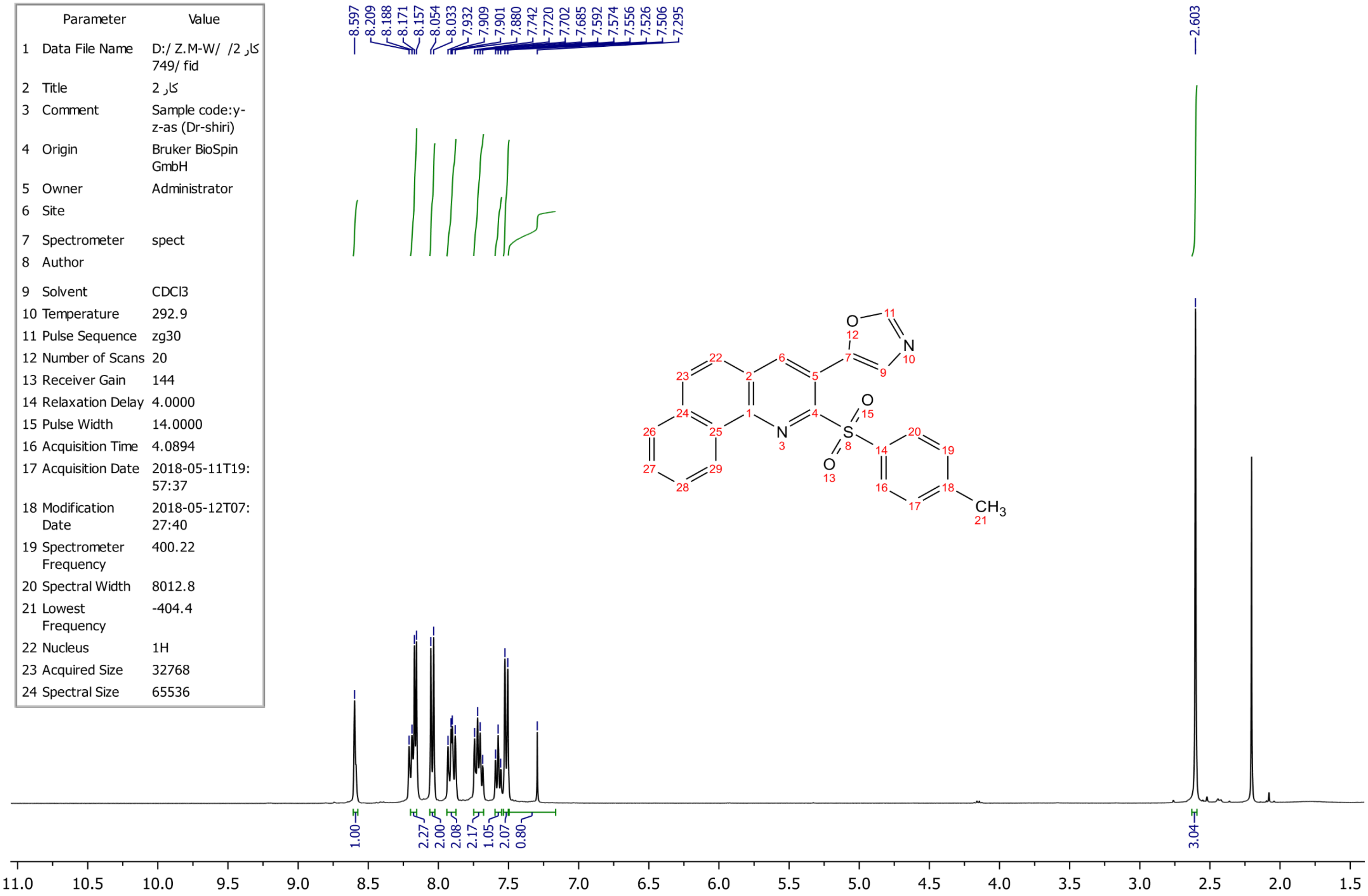

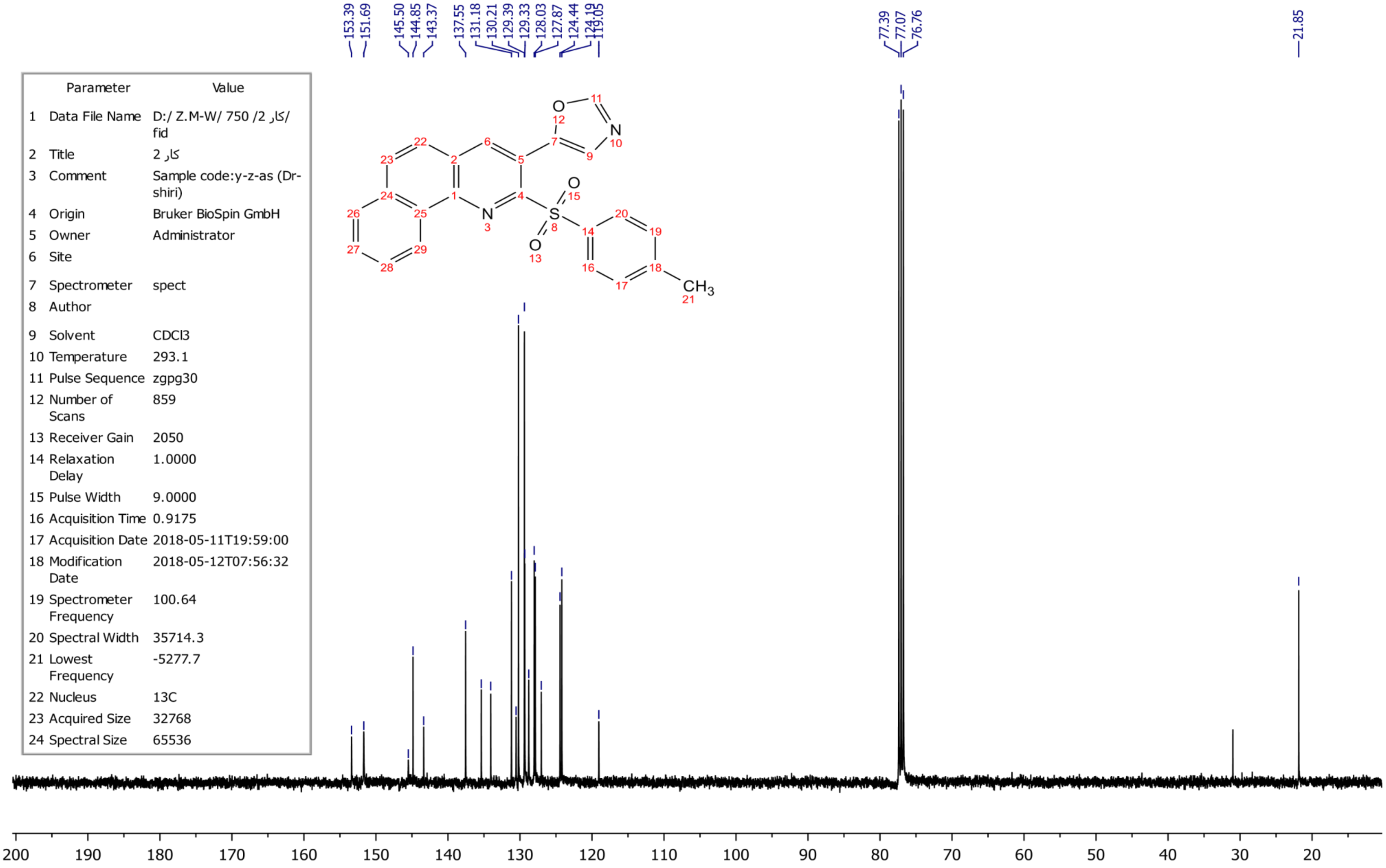

	Parameter	Value
1	Data File Name	D:/ Z.M-W/ 750 /2 کار/ fid
2	Title	2 کار
3	Comment	Sample code:y-z-as (Dr-shiri)
4	Origin	Bruker BioSpin GmbH
5	Owner	Administrator
6	Site	
7	Spectrometer	spect
8	Author	
9	Solvent	CDCl3
10	Temperature	293.1
11	Pulse Sequence	zgpg30
12	Number of Scans	859
13	Receiver Gain	2050
14	Relaxation Delay	1.0000
15	Pulse Width	9.0000
16	Acquisition Time	0.9175
17	Acquisition Date	2018-05-11T19:59:00
18	Modification Date	2018-05-12T07:56:32
19	Spectrometer Frequency	100.64
20	Spectral Width	35714.3
21	Lowest Frequency	-5277.7
22	Nucleus	13C
23	Acquired Size	32768
24	Spectral Size	65536
